# Pseudouridine synthase PUS1 and initiation factor mtIF2 are human mitoribosomal small subunit assembly factors

**DOI:** 10.1038/s41467-026-74700-x

**Published:** 2026-06-24

**Authors:** Vivek Singh, Dmitrii Shiriaev, Lorina Bilalli, Anas Khawaja, Joanna Rorbach

**Affiliations:** Department of Medical Biochemistry and Biophysics, Karolinska Institutet, Stockholm, Sweden

**Keywords:** Cryoelectron microscopy, Ribosome

## Abstract

Assembly of the mitochondrial ribosome (mitoribosome) is a crucial step in mitochondrial gene expression. This process facilitates mitochondrial translation, which produces essential subunits of the oxidative phosphorylation machinery—the cell’s primary energy-producing machinery. Disruptions in mitoribosome assembly can lead to severe human diseases. Given its fundamental importance, detailed structural analysis of mitoribosome assembly pathways is essential for advancing our understanding of mitochondrial function in both health and disease. In this study, we characterize twelve distinct assembly states of the mitoribosomal small subunit (mtSSU) isolated from human cells. Our findings reveal the intricate details of the final maturation stages of the mtSSU platform, decoding center, and the 3’-end of 12S rRNA. This process is governed by coordinated actions of assembly factors that ensure precise, stepwise rRNA folding and the integration of mitoribosomal proteins into the developing subunit. Our approach identifies pseudouridine synthase PUS1 and initiation factor mtIF2 as assembly factors, expanding their known roles beyond mt-tRNA maturation and translation, respectively. In addition, the identified assembly intermediates provide insight into the modular nature of mtSSU biogenesis in mitochondria and further link late-stage assembly to the acquisition of translational competence.

## Abstract (web summary)

Cellular energy production depends on mitochondrial translation. Here, using cryoEM, the authors describe 12 assembly states of the human mitoribosomal small subunit and identify PUS1 and mtIF2 as mitoribosome assembly factors.

## Introduction

Mammalian mitoribosomes have evolved to produce 13 hydrophobic membrane proteins - components of the oxidative phosphorylation system - encoded by mitochondrial DNA. These molecular machines are composed of two subunits - small (mtSSU), responsible for the mRNA reading by corresponding tRNA pairing within its decoding centre, and large (mtLSU), mediating the protein chain elongation inside its catalytic core of peptidyl transferase centre. Each subunit is made of an rRNA strand (12S and 16S for mtSSU and mtLSU) and a number of mitoribosomal proteins (MRPs), with mtLSU additionally having incorporated mt-tRNA^Val^ or mt-tRNA^Phe^ as its integral part^1–3^.

Despite the basic features and functionality of the mitoribosome being similar to those of its counterparts from bacterial and eukaryotic cytosolic systems, the distinct environment of the mitochondrial matrix and the specialized role of the mitochondrial translation machinery have left a unique print on the biogenesis aspects of the mitoribosomes. Mammalian mitochondrial rRNAs, 12S and 16S, have been significantly shortened throughout their evolution. They are transcribed from the mitochondrial genome and sequentially covered by a network of MRPs encoded by nuclear DNA and imported into the organelle. Many universal ribosomal proteins have mitochondria-specific terminal extensions, and almost half of the MRPs are unique to mitochondrial systems only. The proteins join the immature subunits in a coordinated manner with the help of specialized assembly factors, which mediate rRNA folding by their modifications or act as sequestering factors ensuring correct maturation of the target ribosomal regions.

Recent studies of mitoribosome small subunit (mtSSU) assembly in mammals have begun to uncover the details of the process. Kinetic findings^4,5^ suggest that mtSSU maturation occurs co-transcriptionally. MRPs initially bind to the outer surface of the growing mtSSU, away from the intersubunit interface. This binding sequentially facilitates the formation of the primary folds in the lower body, head, and central region of the mtSSU. During the later stages, MRPs join closer to the intersubunit interface, binding at the head and body of the mtSSU. Additional insights have been gained from cryo-electron microscopy (cryo-EM) analyses of late-stage assembly intermediates, where the majority of MRPs are already positioned within the subunit. The study by Harper et al.^6^ captured the folding of 12S rRNA helices 27, 44, 45 (h27, h44, h45) within the mtSSU body, mediated by biogenesis factors GTPase NOA1 and methyltransferase TFB1M. This is followed by the head and platform maturation, where the subset of assembly factors: MCAT, METTL17, ERAL1, and RBFA contribute to the correct rRNA folding, leading to the decoding centre assembly. During the later stages, revealed by Itoh et al.^7^, the platform region becomes fully mature with the help of methyltransferase METTL15, which is then replaced by mtIF3, serving both as an assembly and initiation factor. The last MRP to bind is mS37, which substitutes RBFA, and the subunit becomes ready for the initiation mediated by mtIF2 and mtIF3^7,8^. Together, these studies suggest a model in which the mtSSU body matures relatively early, followed by gradual assembly of the head and platform. However, the earliest intermediate reported by Itoh et al.^7^, features a mature mtSSU head while TFB1M remains bound to an immature mtSSU body, raising the possibility that assembly proceeds modularly, with distinct regions maturing independently. Alternative assembly pathways have also been described in bacteria, supporting the idea that mtSSU biogenesis may not follow a single linear route^9–13^. Recent work using bioinformatics analysis together with insights from the mitoribosome assembly in other eukaryotes suggests a more elaborate maturation process involving an interplay between assembly factors that are yet to be characterized in human mitochondria^14,15^.

To gain deeper insights into mtSSU biogenesis, in this manuscript, we resolved the structures of twelve mtSSU assembly intermediates and translation initiation states, complementing and expanding the known patterns of mtSSU biogenesis. Our analyses (1) identify PUS1 as an assembly factor involved in the folding of h44; (2) elucidate functions of mito-specific elements of previously reported assembly factors, namely, METTL15 and NOA1; and (3) highlight the crosstalk between mtSSU assembly and translation initiation mediated by METTL15 and mtIF2.

## Results

### Structure determination of mtSSU intermediates

To characterize mtSSU assembly intermediates, we collected two independent single-particle cryo-EM datasets. The first dataset (“RCC1L”) was collected from the interactome of FLAG-tagged RCC1L, a putative guanine nucleotide exchange factor. Overexpression of its isoform, RCC1L^v1^, has previously been shown to result in mild accumulation of mtSSU intermediates despite a lack of direct binding^16^. The second dataset (“NOA1”) was obtained from the purified substrates of FLAG-tagged mitochondrial GTPase NOA1, a protein that binds the earliest small subunit biogenesis stage described recently by structural approaches^6^. Thus, the two model systems employed here provide independent and complementary approaches for evaluating the mtSSU assembly pathway. Cryo-EM data processing details can be found in Supplementary Fig. 1 and Supplementary Table 1. Although we employed RCC1L FLAG-tagged for affinity purification, the factor itself was not observed in any of the reconstructed states, likely due to the dynamic nature of its interaction with the mitoribosome assembly intermediates. Particles corresponding to the mtSSU, mtLSU, and monosome were detected in both datasets (Supplementary Fig. 1); however, since the cryo-EM datasets showed greater enrichment of mtSSU-associated particles than mtLSU-associated particles, further classifications and structural analyses were restricted to the mtSSU and its assembly intermediates.

**Fig. 1:**
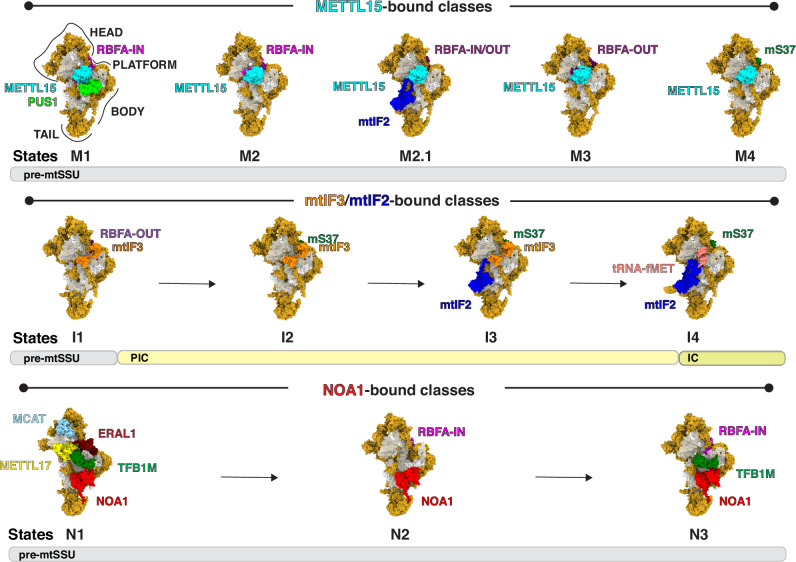
Overview of the mtSSU classes obtained in this study. The atomic models of 12 mtSSU states along the assembly and pre-initiation pathways are represented on the surface. 12S rRNA is colored gray, and mitoribosomal proteins are colored dark yellow, except for mS37, which is colored green. The assembly and initiation factors for all states are illustrated and colored individually. All classes are clustered into three main groups: METTL15-, mtIF3/2-, and NOA1-bound complexes. The color-coded bar at the bottom indicates pre-mtSSU states (gray), pre-initiation (PIC) (yellow), and initiation complex (IC) (olive).

To resolve the consensus map of mtSSU into homogeneous individual states, we performed a series of focussed 3D classifications with signal subtraction, which resulted in 12 classes (Fig. 1; Supplementary Fig. 1). These could be grouped into three major clusters: METTL15-, mtIF2/mtIF3-, and NOA1-bound particles.

The classes with METTL15 consisted of four intermediates (found both in “RCC1L” and “NOA1” datasets): (i) with pseudouridine synthase PUS1 and RBFA in its RBFA-IN conformation (M1); (ii, iii) with RBFA bound in two distinct conformations, RBFA-IN and RBFA-OUT (M2 and M3); (iv) with a translation initiation factor mtIF2 and RBFA (mixed conformations) (M2.1); (v) finally a state resembling the mature mtSSU (M4) with RBFA replaced by the site-specific protein mS37. The mtIF2/3-bound states (found in “RCC1L” and “NOA1” datasets) contained four classes: (i) mtIF3-RBFA-OUT (I1); (ii) mtIF3-mS37 (I2; known as preinitiation complex 1, or mtPIC-1); (iii) mtIF3-mS37-mtIF2 (I3; mtPIC-2); and (iv) initiation complex mS37-mtIF2 with P-site bound fMet-tRNA^fMet^ (I4) (Fig. 1; Supplementary Fig 1). Finally, three classes (found exclusively in the “NOA1” dataset) represented NOA1-bound mtSSU additionally bound by: (i) METTL17, MCAT (head), TFB1M (body), and ERAL1 (platform) (State N1); (ii) RBFA (at the platform) (N2); (iii) TFB1M and RBFA (N3).

### PUS1 acts as an mtSSU assembly factor facilitating h44 accommodation

**Fig. 2:**
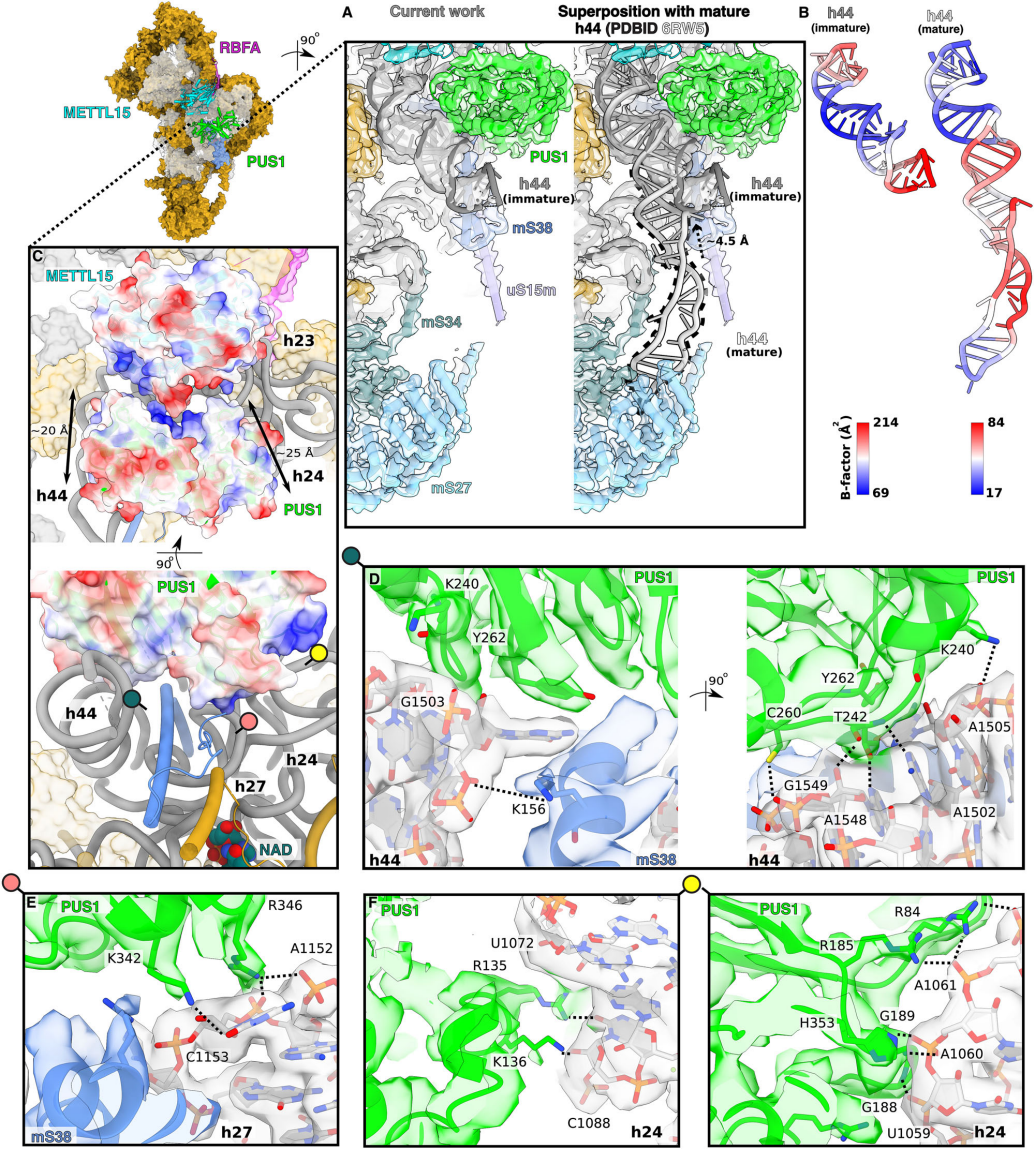
Role of PUS1 in mtSSU assembly. **A** Inset shows the model of mtSSU assembly intermediate bound to METTL15 and PUS1. Zoom-in panel, on the left shows PUS1 bound to immature h44 together with the density map (colored by zone). Mito-specific protein elements (mS38, uS15m N-ter, mS34, and mS27) are shown in shades of blue. Zoom-in panel right shows superposition with mature h44 (from PDBID 6RW5) (marked by a dotted line). **B** Models of h44 from current work (immature) and PDBID 6RW5 (mature) colored by *B*-factor. Respective scales are shown below each model. **C** The zoom-in panel shows the columbic surface of METTL15 and PUS1. Contact of PUS1 with rRNA helices h24 and h44 is indicated by arrows. **D**, **E** Zoom-in of the interface between PUS1, h24, h27, h44 and mS38. Interaction sites between PUS1- **D** h44, **E** h27, and **F** h24 (marked as pale green, salmon, and yellow pins, respectively) are shown together with the density map (colored surface).

The decoding center of SSU is highly conserved and central for translation. The rRNA helix h44 contributes the conserved residues A1556 and A1557 involved in decoding, and together with rRNA linkers: h28-h44 and h44-h45 loops form a component of the A-site and the P-site^17^. Correct accommodation of h44 is therefore a prerequisite for decoding center maturation. METTL15 methylates N4 of C1486 of h44 at the decoding center and is required for mtSSU biogenesis^18,19^. In this work, we report four distinct METTL15-bound mtSSU assembly states together with assembly factor RBFA (IN, OUT, and mixed conformations) with progressively maturing rRNA. Finally, we observe one mature state with mS37 in lieu of RBFA in mtSSU-head (Fig. 1). Of these, the earliest state uniquely features an unaccommodated h44 in an otherwise mature mtSSU-body. Such an immature h44, not observed previously, could only be modelled up to base-pair 1512:1537. The remaining h44 (residues 1513-1536) is disordered in our structure (Fig. 2A, B; Supplementary Fig. 2A, B). We find a large unidentified density atop the distorted h44, which could be unambiguously assigned to a pseudouridine synthase, PUS1 (Fig. 2). PUS1, a human mitochondrial homolog to bacterial TruB, however, has not been known to have a role in ribosome assembly; instead, it has been reported to modify a wide range of mt-tRNA and cytosolic mRNA/tRNA substrates^20–22^. In this complex, PUS1 and METTL15 bind in close proximity and in a complementary fashion (Fig. 2C), forming a large basic surface to scaffold the maturing rRNA (Fig. 2C; Supplementary Fig. 2A). A potential role for an additional assembly factor for mammalian mitoribosomal h44 maturation has been highlighted previously based on the occurrence of polymorphisms in this region of h44 leading to structural variability which could potentially impact h44 folding^15^. The interface between PUS1 and mtSSU is characterized by three prominent regions of contact: (1) ~ 20 Å along the stem of h44, (2) h27 loop where mS38 is docked, and (3) ~ 25 Å along the stem of h24 that contributes to METTL15 binding (Fig. 2A-E).

Interface 1 is marked by the interaction between PUS1 and a partially ordered h44 (Fig. 2A–C). We investigated the structural basis for the observed unaccommodated conformation of h44. B-factor analysis indicates an increase in flexibility around the middle of h44 in mature mtSSU (PDBID 6RW5)^7^, which roughly coincides with the point where h44 is disordered in our structure (Fig. 2A, B). Compared to *E.coli* (PDB ID 9GUQ), h44 in human mtSSU has reduced base-pairing in the stem that causes local melting. Ribosomal protein bS20, which stabilizes the corresponding region of h44 is absent^17^. Finally, close to the decoding center, base-pairing residues in bacteria are replaced by non-base-pairing residues in human mitoribosome, ie, 1491 G:1409 C (*E. coli*)>1556 C:1493 C (human mtSSU) and 1490U:1410 A (*E. coli*)>1555 A:1494 C (human mtSSU), which further contributes to the flexibility of mtSSU h44 (Supplementary Fig. 2C, D). Therefore, in mature mtSSU, h44 requires additional stabilization by mito-specific protein elements, namely the C-terminal helix of uS15m and the N-terminal helix of mS34, and it is additionally docked into a cleft formed by mito-specific protein mS27 in the mtSSU-tail (Fig. 2A; Supplementary Fig. 2C)^17^. The increased flexibility of h44, coupled with the necessity to dock into a rather narrow cleft of mS27, poses a unique challenge for the maturation of h44 and may be further facilitated by PUS1. PUS1 scaffolds h44 such that it is shifted away from the mtSSU-tail and towards the decoding center by one residue length ~4.5 Å (Fig. 2A), resulting in a kink of h44 that coincides with disrupted base-pairing 1556 C:1493 C and 1555 A:1494 C near the decoding center (Fig. 2A; Supplementary Fig. 2C, E, F). Further, G1503 is flipped out of the helix into a small pocket formed by PUS1 and mS38, where this conformation is stabilized by side-chain and backbone interactions (Fig. 2C, D; Supplementary Fig. 2E). The interactions with flipped G1503 together with additional hydrogen bonds on either side, appear to stabilize the interaction of h44 with PUS1, holding h44 retracted away from the mtSSU-tail (Fig. 2C, D; Supplementary Fig. 2C). This retraction of h44 potentially lifts the steric clash between h44 and mS27 as it docks into the mS27 cleft of mtSSU-tail. Interface 2 is marked by a juxtaposition of PUS1, h44, and h27, forming a tunnel-like structure that accommodates mS38 (Fig. 2C, E). Previous work suggests that mS38 tends to be flexible and its recruitment and stabilization are associated with the folding of h44^6,7^. Our structure shows that PUS1 likely serves as a scaffolding factor to stabilize this region in the absence of a folded h44 and facilitate the accommodation of mito-specific protein mS38 through interfaces 1 and 2.

Interface 3 of PUS1 stabilizes h24 in its mature conformation by providing a series of H-bonds and electrostatic interactions (Fig. 2C, F), accommodating the curvature of h24 stem. The loop of h24 together with the base of h44 forms the platform for METTL15 binding (Fig. 2C, 2A)^6,7^.

In the context of the mtSSU assembly intermediate, the binding interface of PUS1 is primarily formed by rRNA. However, the way PUS1 binds in this mode prevents it from performing its usual pseudouridine synthase function. This is because the active catalytic pocket is facing away from the rRNA and is blocked by METTL15 (Supplementary Fig. 2G, H). Also, the residues of PUS1 contributing to mtSSU binding are spatially and structurally separated from its canonical catalytic site (Supplementary Fig. 2H).

PUS1 partly shares its rRNA binding interface with methyl transferase TFB1M. Previous work suggests TFB1M to play a scaffolding role for the stabilization of rRNA h44 and h23-24 in addition to its enzymatic function of methylating h45^6,7^. However, TFB1M poses a clash with METTL15 whose binding is required for eventual decoding center maturation (Supplementary Fig. 3). PUS1 potentially takes over the scaffolding function of TFB1M, with the N-terminal domain of PUS1 superposes with the C-terminal domain of TFB1M to stabilize h23-h24 (Supplementary Fig. 3). Meanwhile, the N-terminal domain of PUS1 is shifted away to scaffold h44, which not only is compatible with but also facilitates METTL15 binding (Fig. 2C; Supplementary Figs. 2A; 3). Indeed, PUS1-METTL15 together scaffold rRNA over an interface of 1722 Å^2^ which is comparable to TFB1M-rRNA interface of 2144 Å^2^, while PUS1 alone has an rRNA binding surface area of 1018 Å^2^.

While PUS1-bound mtSSU was observed in both independent datasets, “NOA1” and “RCC1L”, strongly suggesting its role in mtSSU biogenesis, we sought to further validate this finding. We performed reciprocal immunoprecipitations using PUS1 as bait, where we found components of mtSSU, but not mtLSU, in the elution fraction (Supplementary Fig. 4). Next, to confirm that PUS1-FLAG co-purifies specifically with mtSSU assembly intermediates rather than individual small subunit components or the ribosome, we subjected the IP elution fraction to sucrose gradient separation. The mtSSU proteins pulled down with PUS1 migrated within fractions corresponding to the free small subunit. Notably, mS37—the last MRP to join the small subunit^7^ and absent in the structurally captured intermediate—was also absent in the elution fraction, indicating that PUS1 preferentially binds immature mtSSU. In contrast, free mtLSU and monosomes were significantly less enriched than the small subunit, highlighting the specificity of PUS1 binding to mtSSU (Supplementary Fig. 4).

### METTL15-mediated 3´-end rRNA maturation and translation initiation

**Fig. 3:**
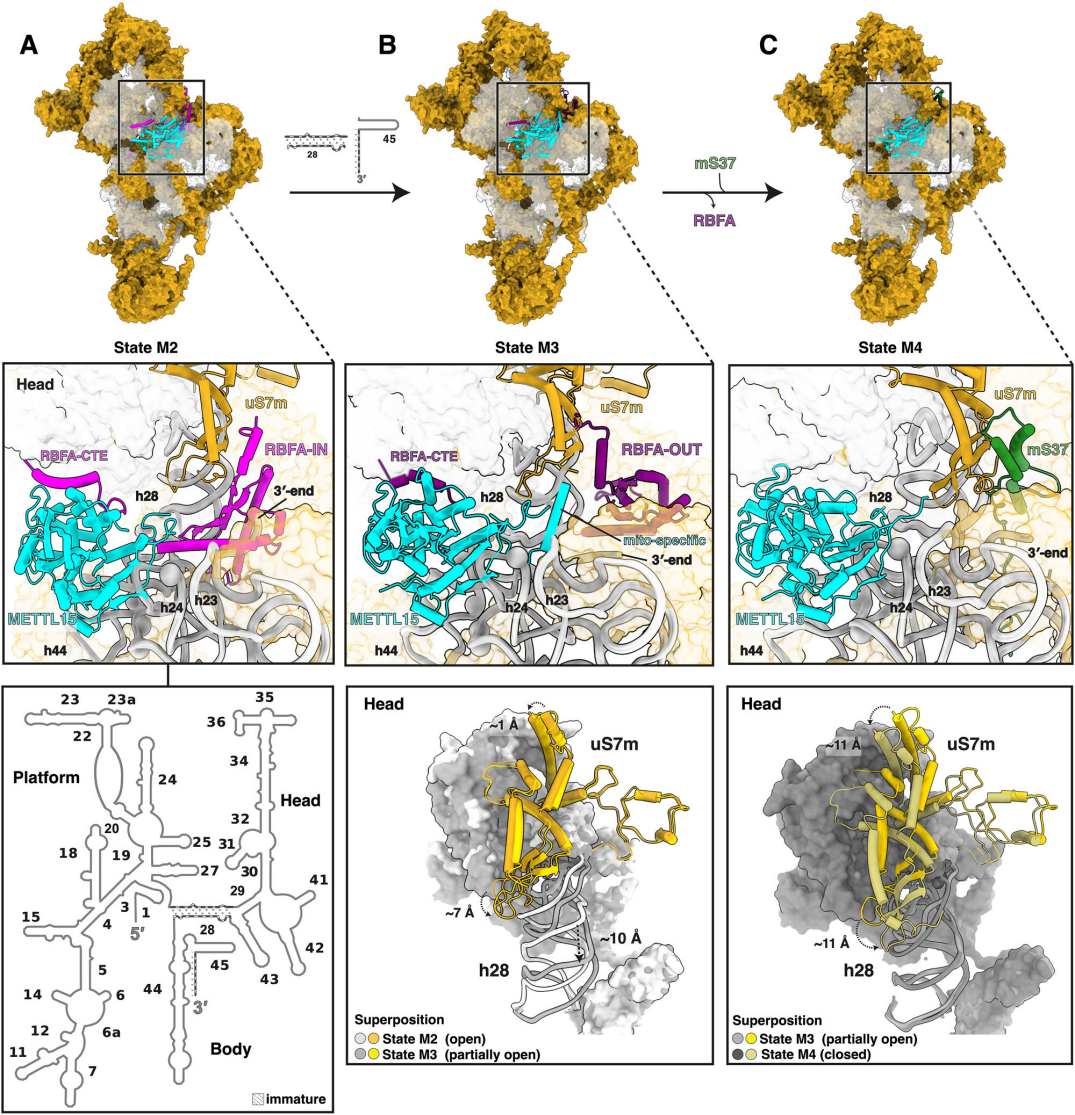
Structures of METTL15-bound mtSSU assembly intermediates. Models of METTL15-bound mtSSU assembly intermediates, M2-M4 (top), show pathways for maturation of rRNA (white transparent surface) and ribosomal proteins (gold surface). Association and dissociation of assembly factors (cartoon) and folding of rRNA are schematically depicted on connecting arrows. **A** Zoom-in panels (middle) show METTL15 (cyan) sequesters h24 and RBFA-IN (magenta) stretches across h28 occupying the exit channel. (Bottom) 2D representation of 12S rRNA in state M2 to highlight immature regions (dashed lines). Secondary structure templates were adapted from Ribovision suite^49^ and further modified for this study. **B** Zoom-in panels (middle) show ordering of METTL15 mito-specific insertion and displacement of RBFA from IN towards OUT (purple). (Bottom) superposition of state M2 and M3 illustrating the shift of h28, uS7m and head rotation (rRNA). The color code for each state is depicted alongside. **C** Zoom-in panels (middle) show mS37 (green) replacing RBFA. (Bottom) superposition of state M3 and M4, showing the overall shift of h28, uS7m and head rotation (rRNA). The color code for each state is depicted in the panel alongside.

As PUS1 dissociates, the subsequent METTL15-bound assembly intermediates (M2-M4) exhibit an accommodated h44, a completely matured decoding center, with the h28-h44 and h44-h45 linkers fully ordered. These assembly states contribute to the platform repositioning and folding of the 3′- end rRNA. The M2 state contains RBFA-IN that occupies the core of the mtSSU platform in the mRNA exit channel, acting as a wedge that restricts the movement of h23-24, inhibiting premature folding of h28 and 3′-end of the rRNA. RBFA packs against almost the entire length of h28 forming an interface of 368 Å^2^, which ensures the restricted movement of h28, keeping it immature, which correlates with the head rotation towards the A-site (Fig. 3A). Additionally, RBFA sequesters the 3′-end of the rRNA (nt. 1598-1601) as it localizes near h36 (~9 Å) in the head domain (Fig. 3A, Supplementary Fig. 5A). METTL15 also participates in restricting the movement of the platform by sequestering the tip of h24 via its MTase domain (Fig. 3A).

In the following state, M3, the mito-specific helix of METTL15 is inserted at the mRNA exit channel, effectively displacing the RBFA’s N-terminal domain (NTD) to the peripheral region of the mtSSU platform (RBFA-OUT) (Fig. 3B). Relocation of RBFA to its OUT conformation disrupts its contacts with h28, which leads to h28 transition to a near-mature state as the head domain compacts ~10 Å towards the E-site. Consequently, uS7m is shifted downward by approximately 7 Å from its earlier M2 state (Fig. 3B, bottom), exposing the P-site. At this point, METTL15 mito-specific insertion also serves as a spacer between the platform region and the head domain, limiting the movement of h23-h24 and β-hairpin of uS7m. This keeps the head in a partially open conformation, facilitating maturation of h28 (Fig. 3B). Meanwhile, the rRNA 3′-end is incorporated in its near-mature conformation (Supplementary Fig. 5B). The next state was recently reported by Itoh et al.^7^ (PDBID 7PNZ) and potentially constitutes the final assembly intermediate that contains both METLL15 and RBFA-OUT. In this state, METTL15 loses contact with the h24, which causes the entire platform to shift (~ 5 Å) towards the head domain, sterically hindering the mito-specific insertion from the exit channel (Supplementary Fig. 5C)^7^. Taken together, the subtle movement of METTL15 on the mtSSU through all observed RBFA-bound states (~ 9 Å) correlates with shifting of the platform (~11 Å), suggesting that METTL15 binding, coupled with decoding center maturation, controls the stepwise repositioning of the platform to its final state (Supplementary Fig. 5D).

As METTL15 remains associated with the mtSSU, the next state, M4, guides the final architecture of the 3´-end rRNA. The departure of RBFA and the removal of the mito-specific insertion of METTL15 allows the uS7m β-hairpin to be more flexible, causing the head to adopt a closed state (Fig. 3C). The interplay between mS37 binding and closed head conformation facilitates the complete folding of the 3´-end rRNA (Fig. 3C and Supplementary Fig. 5B, right panel).

With the incorporation of the final mitoribosomal protein mS37, state M4 represents a mature mtSSU. This herein observed state complements the previously reported translation pre-initiation complex containing mtIF3 (State I2)^7,8^, repurposing METTL15 as a potential pre-initiation factor, which guides preparation of mtSSU to translation initiation in a similar manner as mtIF3.

The presence of both I2 and M4 in our dataset (Fig. 1) suggests the coexistence of parallel assembly routes, where either METTL15 is replaced by mtIF3 or persists, with both circumstances leading to translation-competent mtSSU. Both mtIF3-CTD and METTL15 stretch over a similar surface area on h24 and 44 (~36 Å) of the rRNA (Supplementary Fig. 5E), which correlates with the stabilization of the platform interface prior to the initiation complex formation (Fig. 6), implying overlapping functions of mtIF3 and METTL15. This observation aligns with previous studies demonstrating that in human cells depleted of mtIF3, translation of mitochondrial leaderless transcripts is not affected, indicating that translation-competent ribosomes can be formed without mtIF3^23,24^. Alternatively, these intermediates (states M4 and I2) may reflect a regulatory quality-control mechanism in which METTL15 and mtIF3 act sequentially to assess and stabilize rRNA elements of the platform region before progression to the initiation complex. Such a mechanism would ensure that only properly folded and functionally competent mtSSU particles proceed, thereby maintaining the fidelity and efficiency of translation.

### mtIF2 role in mtSSU assembly

**Fig. 4:**
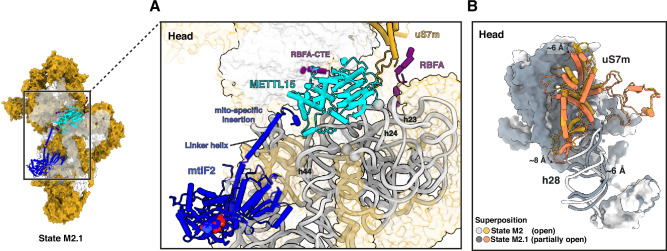
Binding of mtIF2 during mtSSU assembly. **A** Model of mtSSU assembly intermediate bound to mtIF2 (blue), METTL15 (cyan) and RBFA (purple). Zoom-in panels (left) show the overall position of mtIF2 on the mtSSU as it comes in close contact to METTL15 via its mito-specific insertion. RBFA is in transition between the IN and OUT states. **B** Superposition of state M2 and M2.1 illustrating the shift of h28, uS7m and head rotation. The color code for each state is depicted alongside.

Our classification of cryo-EM particle images resulted in the identification of an mtIF2-bound mtSSU assembly intermediate in complex with METTL15 and RBFA-IN/OUT (Fig. 4A). This class, M2.1, comprises only ~1% of the particles (in both “RCC1L” and “NOA1” datasets), suggesting a transient nature of this state. mtIF2, a translation initiation factor, blocks the A-site by interacting with h18, h44, and uS12m through its mito-specific insertion and linker helix. In the M2.1 assembly intermediate, we observe the same domain organization of the mtIF2 on mtSSU as during translation initiation^8^; however, domain IV, which interacts with the fMet-tRNA^Met^ during translation initiation, is considerably flexible. Moreover, the segment of mtIF2 insertion and helix (501-509 aa), which contacts h44, is disordered and lies in close proximity to the METTL15 helical domain (174-293 aa). The rRNA folding status of the mtIF2-assembly intermediate resembles that of state M2, characterized by an immature 3′-end (Fig. 4A). However, the head swivels in a partially open conformation, adopting an intermediate position that is more similar to what is observed in the RBFA-OUT (M3) state. This midway conformation of the head correlates with the transition state of RBFA from IN towards OUT, which also stabilizes h28 in its near-mature conformation (Fig. 4B). The mtIF2 insertion keeps the head and body modules apart, subsequently driving the installation of METTL15 mito-specific helix, which eventually evacuates the RBFA-NTD to the peripheral exit channel (state M3, Fig. 3B, middle panel).

To confirm the specificity of the interaction of mtIF2 with the METTL15-bound assembly intermediate, we performed a reciprocal immunoprecipitation (IP) experiment using FLAG-tagged mtIF2 as bait. Western blot analysis revealed that METTL15 is indeed enriched in the mtIF2-IP (Supplementary Fig. 6), confirming the structural observation and supporting the hypothesis of mtIF2 involvement in mtSSU assembly. These findings thus expand the body of evidence of initiation factors acting at the crosstalk of the ribosome maturation and translation^7,14^.

### NOA1-TFB1M interplay regulates mtSSU-body maturation

**Fig. 5:**
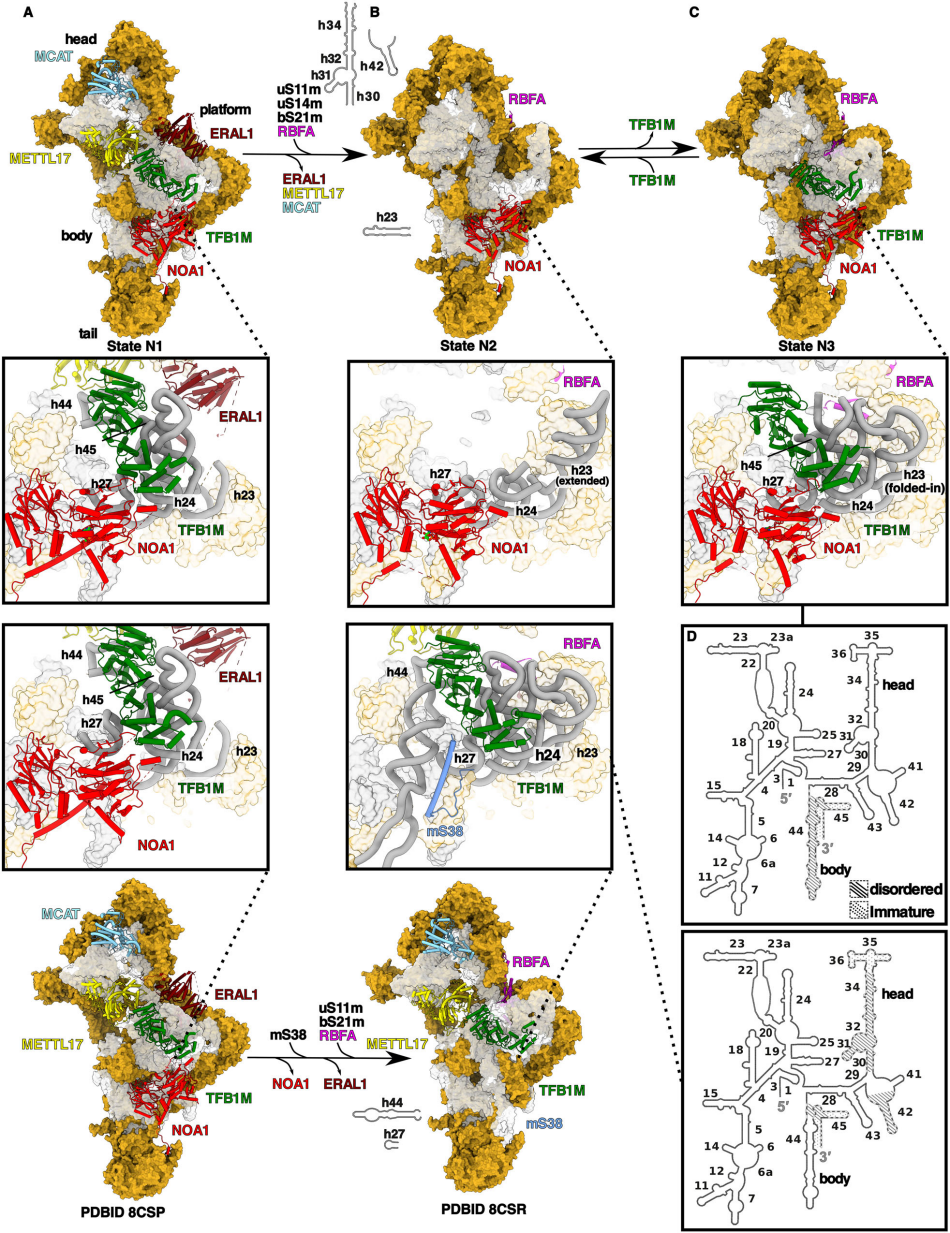
NOA1 bound mtSSU assembly intermediates suggest possibility of an alternative assembly pathway. **A**–**C** Models of mtSSU assembly intermediates from current work, N1-N3 (top), and published work, PDBID 8CSP and 8CSQ (bottom)^6^, show alternative pathways for the maturation of rRNA (white transparent surface) and ribosomal proteins (gold surface). Association and dissociation of assembly factors (cartoon), recruitment of proteins, and folding of rRNA are schematically depicted on connecting arrows. Zoom-in panels (middle) show rearrangements and maturation of rRNA helices associated with TFB1M (green) and NOA1 (red). **D** 2D representation of 12S rRNA in state N3 and PDBID 8CSQ^6^ to highlight immature (dashed lines) and disordered regions (solid lines). Secondary structure templates were adapted from Ribovision suite^49^ and further modified for this study.

The earliest mtSSU assembly intermediate (PDBID 8CSP^6^) reported so far revealed that the GTPase NOA1, together with TFB1M, forms a steric barrier to premature folding of h44, h27 and recruitment of mS38. The dissociation of NOA1 was proposed to be necessary and sufficient to allow maturation/recruitment of these elements, which subsequently occurs in a single step (PDBID 8CSQ^6^). Furthermore, in the proposed assembly pathway, mtSSU body matures earlier than the mtSSU head^6^. In the current work, state M1 shows a nearly mature head but an immature body carrying a partly unfolded h44 bound by PUS1 (while h27 and mS38 are accommodated). This alludes to a more complex pathway for h44-h27 maturation and mS38 recruitment. Therefore, to further explore the details of body folding, we focused our analysis on NOA1-bound states found in “NOA1” dataset (Figs. 1, 5). Focused 3D classification resulted in three distinct intermediates: N1-N3 with NOA1-GDP bound to a progressively maturing mtSSU body while the head is largely mature. Thus, states N1-N3 together with state M1, highlight the stepwise maturation of mtSSU-body, which lags behind head maturation, as detailed below.

Compositionally, our earliest assembly state, N1, closely resembles the previously reported earliest assembly state (PDBID 8CSP^6^). However, conformationally, there are subtle differences, namely the head is more compacted in N1, with a 12-14 Å movement. This brings the decoding center closer to the mature state and pushes TFB1M ~ 5 Å closer to NOA1, effectively shifting h27 ~ 14 Å into an intermediate between the reported immature and its fully folded conformations (Supplementary Fig. 7A). Together, these differences place state N1 later in the assembly timeline compared to 8CSP^6^, which then moves into a pathway of events - from states N1 to N3, characterized by a rapid maturation of the head, while the body (h44) and platform (h28-h44 h44-h45 linkers and 3′-end) remain immature (Fig. 5).

State N2 is characterized by a near-complete maturation of the head with the recruitment of uS14m and maturation of rRNA h30-32, h34, and h42, while the assembly factors METTL17 and MCAT depart. At the platform, uS11m and bS21m bind as ERAL1 is displaced by the chaperone RBFA. Finally, at the largely immature body, NOA1 remains bound while TFB1M dissociates, resulting in two distinct events: (i) lifting of NOA1-TFB1M steric barrier to h27, allowing it to slide into its mature state (Fig. 5B, Supplementary Fig. 7B); (ii) disorder of rRNA h24, h44, h45, and a rearrangement of h23 into an extended conformation (Fig. 5B).

Notably, TFB1M reassociates (state N3), causing a partial re-stabilization of h24 in its near-mature conformation, and rearrangement of h23 into its final, “folded-in” position (Fig. 5C). Such a reassociation of TFB1M leads to the full accommodation of h45; the mature conformation of h27 is retained, while h44 is now the only rRNA element of the mtSSU body that remains disordered (Fig. 5C, D).

This data shows that NOA1 dissociation is not necessary for h27 maturation, as it can also be achieved by TFB1M dissociation. This route further separates the maturation of h27 from the ordering of h44 and the recruitment of mS38, distinguishing these as independent steps rather than a single-step event as previously reported^6^ (Fig. 5A–C, Supplementary Fig. 7B). This is indeed consistent with state M1, wherein h27 is mature while h44 is still disordered. Potentially, dissociation of NOA1 is not sufficient for h44 maturation, which occurs as a distinct step following the accommodation of mS38.

Further, the C-terminal helix of mS38 is stacked with the mito-specific C-terminal helix of uS15m. We observe that the mito-specific N-terminal helix of NOA1 (residues 88-114) that is largely ordered in state N1 becomes partially displaced in states N2-N3. This allows the docking of the C-terminal helix of uS15m, serving as a platform for eventual recruitment of mS38 in state M1 (Supplementary Fig. 7C; Fig. 2). This highlights a human mito-specific mechanism of mtSSU maturation by NOA1. While we could observe the interplay of the N-terminal helix of NOA1 with uS15m, comparison with its bacterial homolog reveals large mito-specific elements, only some of which could be modelled here (Supplementary Fig. 8A), and the role of those mito-specific elements remains to be elucidated.

States N1-N3 and state M1 together reveal a more detailed pathway of mtSSU-body maturation and, in the context of previous work, suggest a modular model for late-stage mtSSU assembly in which different domains can mature semi-independently. Specifically, Harper et al. isolated intermediates using endogenously tagged biogenesis factor, METTL17, whereas distinct mtSSU intermediates (N1-N3) in our dataset were captured following overexpression of NOA1. It is possible that the distinct purification approaches influence the kinetics of mtSSU maturation by stabilizing specific transient intermediates that are not readily detected or not preferentially populated under physiological conditions.

**Fig. 6:**
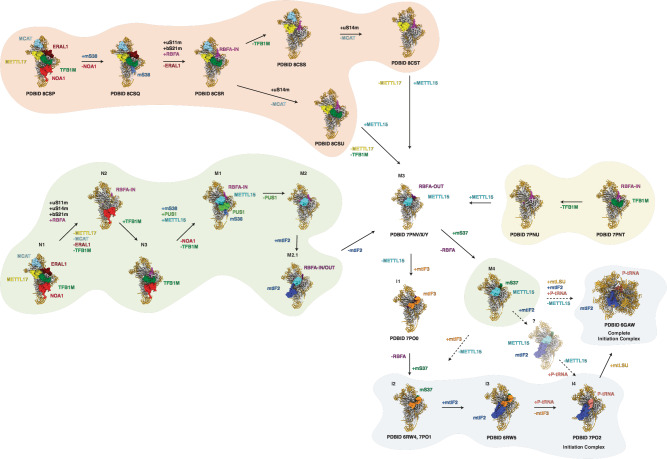
Combined pathways of mammalian mtSSU biogenesis. The schematic models are focused on the arrival of MRPs and the participation of the assembly factors. 12S rRNA is colored gray, mitoribosomal proteins are colored dark yellow, except mS37 and mS38. For the states that were previously published, PDB codes are provided. Three distinct assembly pathways are colored differently: light green background, observed in the current study; light red background, observed by Harper et al.^6^; light yellow background, observed by Itoh et al.^7^. Preinitiation or initiation states are colored with a light blue background and reflect two possible ways to achieve initiation complex: via the mtIF3-pathway (state I2) or via the non-mtIF3 M4 state.

Based on the maturity of rRNA elements (conformation of rRNA helices and chemical modification of its nucleotides), as well as the presence of biogenesis factors within the observed mtSSU assembly intermediates, we organized the classes into a chain of events corresponding to a probable assembly pathway starting from a relatively immature mtSSU (state N1) to the initiation complex (state I4) (Fig. 6).

## Discussion

Ribosome assembly is a complex process involving a series of precise spatiotemporal rRNA-protein binding events. These events result in correctly matured subunits that ensure high translational fidelity, which is essential for proper cellular function. In this study, we have elucidated the final phases of 12S rRNA folding, involving a network of regulatory factors that act in a sequential manner (Fig. 6).

We identified a pseudouridine synthase, PUS1, as a participant in the mtSSU assembly. The repurposing of proteins with other established functions in the maturation process is a well-recognized aspect of mitoribosome biogenesis. Examples include: MCAT, a factor involved in fatty acid metabolism^25^, also contributing to the assembly of the mtSSU head^6^; NSUN4, a methyltransferase that modifies m^5^C1488 of h44 of 12S rRNA and additionally acts as a non-methyltransferase biogenesis factor on mtLSU^26^; mt-EF-Tu, which delivers charged tRNA molecules to the ribosome and participates in mtLSU assembly^27^; mt-ACP, which assists in the large subunit biogenesis while also serving as a fatty acid synthesis scaffold^28^. Likewise, PUS1 emerges as another candidate for such “double agents”, which contributes to the stabilization of 12S rRNA, mS38, and METTL15 binding during assembly, a role independent from its pseudouridinylation activity. Given that PUS1 mutations are associated with mitochondrial pathologies^29,30^, further studies are warranted to explore its function in the context of disease.

An important theme highlighted by our work is the dual functionality of methyl transferases. We show that methyl transferases, METTL15 and TFB1M, in addition to methylating rRNA residues of mtSSU, also fulfill a scaffolding function for the maturing rRNA. We uncover a unique feature of TFB1M involving its dissociation and reassociation thereby regulating the maturation of rRNA h27 and eventual recruitment of mS38 (Fig. 5; Supplementary Fig. 7). It is unclear at what stage METTL15 methylates C1486, however, it is docked through several assembly intermediates up to a mature mtSSU, forming a network of transient interactions with other assembly factors to facilitate (1) the accommodation of h44 aided by PUS1 (Fig. 2; Supplementary Fig. 2); (2) maturation of the decoding center with methylation of C1486 and (3) maturation of the platform in concert with RBFA and mtIF2 resulting in stabilized h23/24 and recruitment of mS37 (Fig. 3). Previously, for mtSSU-head maturation, a key scaffolding function was attributed to methyl transferase, METTL17 that binds h38 during mtSSU assembly and facilitates mtSSU-head compaction^6^. Similarly, for mtLSU maturation, NSUN4 plays a purely scaffolding role together with MTERF4 to facilitate PTC maturation^27,31–34^. Furthermore, it has recently been shown that mtLSU rRNA can reach a near-mature state in the absence of mitochondrial methylation potential, highlighting a non-enzymatic role of methyl transferases^35^. Thus, our data provides evidence and a structural basis that methyl transferases largely do not just play a purely enzymatic role in the methylation of rRNA but also actively scaffold the rRNA and facilitate the binding and function of other factors in the process of human mtSSU biogenesis.

Furthermore, we identified mtSSU intermediate complexes containing mtIF2, a canonical translation initiation factor, underscoring the dual role of this protein. A similar repurposed function of IF2 has been observed in bacteria during the cold shock response^36^. Studies in eukaryotes have also shown the presence of mtIF2 on the mtSSU assembly intermediate in *T. brucei*^14^, highlighting the crosstalk between the process of ribosome assembly and translation.

Together, our findings identify additional assembly factors and illustrate the flexibility of the mitoribosomal small subunit assembly process. This insight is crucial as it broadens our understanding of mitoribosomal biogenesis and translation, highlighting that multiple routes can lead to functional mitoribosomes.

### Limitations of the study

In the current study, we used RCC1L and NOA1 as baits, which enabled the visualization of several transient steps of body and platform maturation that would otherwise be too brief to capture structurally. However, the high levels of overexpression could have affected assembly dynamics and potentially led to off-pathway states. We have therefore focused on the interpretation of the states that were identified in both datasets. Further studies of the assembly intermediates identified only in one dataset (NOA1) will have to be further validated.

## Methods

### Cell lines

The Flp-In T-Rex human embryonic kidney 293 (HEK293) cell line (Invitrogen) was used to produce NOA1::FLAG and mtIF2::FLAG overexpression cell lines. All cell lines used in the study were cultured in DMEM (high glucose, GlutaMAX, sodium pyruvate) (Thermo Fisher) supplemented with 10% (v/v) fetal bovine serum (FBS) (Thermo Fisher), 1× Penicillin/Streptomycin (Thermo Fisher), and 50 µg/ml uridine at 37 °C and 5% CO2 unless stated otherwise. For maintenance of overexpression cell lines, the medium was supplemented with 100 µg/ml blasticidin and 100 µg/ml hygromycin.

### Generation of overexpression cell lines in HEK293

HEK293 cells were transfected into one well of a 6-well culture plate with the pcDNA5/FRT/TO vector carrying C-terminally FLAG-tagged sequences of NOA1, RCC1L (isoform 1), mtIF2, mS27, PUS1, or firefly luciferase carrying mitochondria targeting sequence (mtLuci) as well as the pOG44 construct using Lipofectamine 3000 according to the manufacturer’s instructions. After 24 hours, cells were subjected to selective medium containing 15 μg/ml Blasticidin S and 100 µg/ml Hygromycin B as extra components. 2-3 weeks after the transfection, resistant clones were selected and tested for the inducible overexpression.

### Purification of mitochondria

Cells at the desired confluency were collected and resuspended in an ice-cold hypotonic buffer (0.6 M mannitol, 10 mM Tris-HCl pH 7.5, 1 mM EDTA pH 8, 0.05% bovine serum albumin; approximately 4 ml buffer per 1 ml cell pellet) followed by mitochondrial extraction using dounce-homogenizers and differential centrifugation - at 400 *g* for 5 min at 4 °C to remove non-disrupted cells and debris, and at 11,000 *g* for 10 min at 4 °C to sediment mitochondria. The pellet formed during low-speed centrifugation was subjected to a next round of homogenization, if necessary.

### Purification of NOA1-bound mtSSU intermediates

NOA1 overexpressing HEK293 cells were seeded on eighty 150 mm dishes and induced at the end of the exponential phase with 50 ng/ml doxycycline for 48 hours. The mitochondrial fraction, obtained as in the previous section, was further purified by sucrose cushion (20 mM Tris-HCl, pH 7.5, 1 mM EDTA, 1.0 M and 1.5 M sucrose; centrifuged for 1 h at 25,000 rpm. (SW41 Ti rotor, Beckman Coulter)) and collected as a band located between the two layers of sucrose buffers. Upon washing with 10 mM Tris-HCl pH 7.5 in a 1:1 ratio to reduce sucrose content, the mitochondrial fraction was pelleted, resuspended in 300 µl of freezing buffer (10 mM Tris-HCl pH 7.5, 10 mM KCl, 0.1% bovine serum albumin, 1 mM EDTA, 200 mM trehalose), flash-frozen in liquid nitrogen, and stored at −80 °C until further use.

Mitochondrial fraction was thawed and lysed in 2 ml of lysis buffer (25 mM HEPES-KOH pH 7.5, 25 mM KCl, 20 mM Mg(OAc)2, 2% Triton X-100, 0.1 mM DTT, 1X cOmplete EDTA-free protease inhibitor cocktail (Roche), and 400 U RNase inhibitor (Invitrogen) for 30 min at 4 °C. After centrifugation for 10 minutes at 21,300 *g* at 4 °C, clarified lysate was applied to 100 µl ANTI-FLAG M2 Affinity Gel (Sigma–Aldrich), prewashed with 25 mM HEPES-KOH pH 7.5, 25 mM KCl, 20 mM Mg(OAc)2, and left for incubation for 3 h at 4 °C. Eventually, the gel with bound complexes was washed three times with the washing buffer (25 mM HEPES-KOH pH 7.5, 25 mM KCl, 20 mM Mg(OAc)2, 0.05% β-DDM) and the complexes were eluted by applying 35 µl of the washing buffer containing 0.5 µg/µl 3X FLAG peptide (Sigma) (elution buffer) for 30 minutes at 500 rpm shaking.

### Purification of mtSSU intermediates in the RCC1L-overexpression model

HEK293T cells overexpressing RCC1L were induced with 50 ng/ml doxycycline for 48 hours. The mitochondria were isolated and stored as described in the preceding section. The purified mitochondria were lysed by incubating at 4 °C for 20 min in the lysis buffer (25 mM HEPES-KOH, pH 7.5, 20 mM Mg(OAc)2, 25 mM KCl, 2% (v/v) β-DDM, 0.2 mM dithiothreitol (DTT), 1X cOmplete EDTA-free protease inhibitor cocktail (Roche), 40 U/µl RNase inhibitor (Invitrogen). The lysate was centrifuged at 21,300 g for 10 min at 4 °C, and the supernatant was added to ANTI-FLAG M2 Affinity Gel (Sigma–Aldrich), equilibrated with the wash buffer (25 mM HEPES-KOH pH 7.5, 20 mM Mg(OAc)2, 25 mM KCl, 0.05% β-DDM). After 2 h incubation at 4 °C, the gel was washed with the wash buffer. Next, RCC1L-bound complexes were eluted in 30 µl of the washing buffer with 0.5 µg/µl 3X FLAG peptide (Sigma) (elution buffer) for 30 minutes and 500 rpm shaking at 4 °C.

### Purification of mtIF2-bound mtSSU intermediates

HEK293 cells overexpressing FLAG-tagged mtIF2 or control lines with mS27 and mtLuci were seeded on five 150 mm dishes and induced at the end of the exponential phase with 50 ng/ml doxycycline for 48 hours. mtIF2-bound complexes were extracted similarly to the protocol described in the previous section, except the mitochondria were used fresh, no sucrose cushion was used for further purification, 500 µl of lysis buffer and 20 µl of anti-FLAG gel were used.

### Purification of PUS1-bound mtSSU intermediates

HEK293 cells overexpressing FLAG-tagged PUS1 and the control line (wild-type HEK293) were seeded on ten 150 mm dishes and induced at the end of the exponential phase with 50 ng/ml doxycycline for 48 hours. PUS1-bound complexes were extracted similarly to the protocol described in the previous section, except no sucrose cushion was used for further purification, 30 µl of freezing buffer was used for mitochondria freezing, 800 µl of lysis buffer and 30 µl of anti-FLAG gel were used, and elution was performed twice with 35 µl of the elution buffer. Eluted complexes were separated in a continuous sucrose gradient (10–30% (w/v), 20 mM Tris-HCl pH 7.5, 50 mM KCl, 20 mM MgCl2, 1x PIC) and centrifuged for 2 hours 15 minutes at 39,000 rpm at 4 °C (Beckman Coulter TLS55 rotor). A total of 17 fractions was collected, each with a volume of 100 µl, and 20 µl of each fraction was used for the Western Blot analysis. Fractions 1 and 2, and fractions 16 and 17, were mixed and resolved simultaneously. As a control, 2 µl of input mitochondrial lysate was loaded onto the gel.

### SDS–PAGE and western blot analysis

Samples were resuspended with 1× NuPAGE LDS sample buffer (Thermo Fisher) supplemented with 100 mM DTT, heated for 5 min at 75 °C, and resolved on NuPAGE 4–12% Bis-Tris gels (Thermo Fisher) using NuPAGE 1× MES (Thermo Fisher) running buffer. Proteins were blotted onto PVDF membrane using 40 mM glycine, 50 mM Tris, 20% ethanol, and 0.025% SDS at 4 °C for 90 min at 300 mA. Membranes were blocked for 1 h at room temperature with 5% (w/v) milk in PBS with 0.1% Tween 20 (PBS-T). The blocked membranes were incubated overnight at 4 °C or for 1 h at RT with primary antibodies in PBS-T at the indicated dilutions (Supplementary Table 2). Following incubation, membranes were washed three times with PBS-T and incubated with HRP-conjugated secondary antibodies in PBS-T for 1 h at RT. Membranes were washed three times with PBS-T. Detection was performed using Amersham ECL western blotting detection reagent (GE Healthcare, Amersham). Uncropped blots are depicted in the source data file.

### [35S]-de novo labelling of mitochondrial proteins

Six-well plates with HEK293 cells overexpressing NOA1 or mtLuci were treated with 0.01% (w/v) poly-L-Ornithine in PBS for 1 h at 37 °C, washed three times with PBS and treated with 2 µg/ml laminin in PBS ON at 4 °C. The next day, surfaces were washed three times with PBS and 1 × 10^5^ cells were seeded per well. After 24 hours, cells were induced with 50 ng/ml doxycycline for 48 hours. Afterwards, cells were incubated twice for 5 min at 37 °C in Cys-/Met-free medium (DMEM, high glucose, no glutamine, no methionine, no cysteine, supplemented with 10% dialyzed fetal bovine serum, 1× GlutaMax, and sodium pyruvate) followed by 20 min incubation at 37 °C in Cys-/Met-free medium supplemented with 100 µg/ml emetine to inhibit cytosolic translation. Subsequently, 1 ml Cys-/Met-free medium supplemented with 200 µCi of EasyTag EXPRESS [35S] protein labeling mix (methionine and cysteine) (Perkin Elmer) was added to each dish and incubated for 45 min at 37 °C, 5% CO2. Following labeling, cells were washed three times with 5 ml PBS, harvested by trypsination and consecutive centrifugation (5000 × *g*, 5 min, 4 °C), and stored at −20 °C. Cells were lysed by resuspension in 30 µl PBS supplemented with Complete EDTA-free protease inhibitor cocktail and 50 U Pierce universal nuclease (Thermo Fisher) and application of one freeze-thaw cycle. Protein contents were determined by Pierce BCA assay (Thermo Fisher). 1× NuPAGE LDS sample buffer (Thermo Fisher) was added to 30 µg lysate, and the sample was separated on NuPAGE 4–12 % Bis-Tris mini gels (Thermo Fisher). Coomassie staining was performed using Imperial Protein Stain (Thermo Fisher) according to the manufacturer's suggestions. The gel was fixed in fixing solution (20% methanol, 7% acetic acid, 3% glycerol) for 1 h at RT and vacuum-dried at 65 °C for 2 h. The dried gel was exposed to storage Phosphor screens (Fujifilm) and visualized with Typhoon FLA 7000 Phosphorimager (GE Healthcare). Band intensities were quantified by ImageJ [https://imagej.net/ImageJ].

### Sucrose gradient centrifugation analysis

Mitochondrial pellets were lysed in lysis buffer (10 mM Tris-HCl pH 7.5, 50 mM KCl, 20 mM MgCl2, 1x PIC, 1% Triton X-100) containing freshly added RNase Block and 1 mg of the protein content was loaded onto a continuous sucrose gradient (10–30% (w/v), 20 mM Tris-HCl pH 7.5, 50 mM KCl, 20 mM MgCl2, 1x PIC) and centrifuged for 15 hours at 79,000 x g at 4 °C (Beckman Coulter SW41-Ti rotor). A total of 25 fractions was collected, each with a volume of 450 µl, and 15 µl of each fraction was used for the Western Blot analysis. Fractions 1 and 2, and fractions 18 and 19, were mixed and resolved simultaneously.

### Cryo-EM and image processing

For the NOA1 sample, Cu 300 mesh R 2/2 (Quantifoil Micro Tools GMBH) and Au R 1.3/1.2 300 mesh (Quantifoil Micro Tools GMBH) were used. For RCC1L samples, Cu 300 mesh R 2/2 (Quantifoil Micro Tools GMBH) was used. The grids were coated with 3 nm thick continuous carbon and glow-discharged (15 mA for 2 minutes) using a PELCO easiGlow glow-discharge unit. At 4 °C and 100% humidity, 3 μl of the sample were applied to the grids, which were then blotted (blot time 3 s, blot force 1, waiting time 30 s, 595 filter paper (Ted Pella Inc.) and vitrified in liquid ethane, using a Vitrobot Mk IV (Thermo Fisher Scientific). Grids for the RCC1L sample were frozen with or without the cross-linker 0.8 mM BS3 (Bissulfosuccinimidyl suberate). The sample was incubated with BS3 (11 Å spacer arm) for 30 minutes on ice before grid freezing to prevent degradation of sensitive complexes as described previously^7,35^.

Data were acquired in nanoprobe EFTEM SA mode with a slit width of 10 eV using a K3 Gatan Bioquantum ¨detector. For both datasets of RCC1L (with and without BS3), movies were collected at a magnification of 105 kx (0.825 Å/pix), 40 frames/movie, and a flux of ~1 e/Å^2^/frame for RCC1L data. For NOA1, movies were collected at a magnification of 165 kx (0.505 Å/pix), 40-42 frames/movie, 0.75 electrons/Å^2^ per frame for datasets 1-3 and 0.38 electrons/Å^2^ per frame for dataset 4, respectively.

For NOA1 datasets, motion correction, CTF-estimation, Fourier cropping to 1.01 Å/px for NOA1 data, picking and extraction in a 600 pixel box (size threshold 300 Å, distance threshold 20 Å, using the pre-trained BoxNet2Mask_20180918 model) were performed on-the-fly using Warp 4.0^37^. Only particles from micrographs with an estimated defocus between −0.2 and 3.0 μm were retained for further processing. For RCC1L datasets, the same procedure was applied as above without Fourier cropping (i.e., the original pixel size of 0.825 was retained).

The extracted particles were exported to CryoSPARC^31^ and subjected to 2D classification to discard contaminants. (Supplementary Fig. 1A-C). The mtSSU particles underwent 3D refinement using CryoSPARC^38^ followed by two to three rounds of sequential heterogeneous refinements to remove poorly aligned particles. Well-resolved classes were pooled and subjected to 3D refinement and per particle CTF refinement by CryoSPARC, giving a final particle number of 400 516, 158 970 and ~452 800 mtSSU particles, respectively, from RCC1l (with and without BS3)- and NOA1-datasets.

SSU particle images were then exported to Relion 3.1^39^ or Relion 5.0 for further 3D classification. The particles were subjected to step-wise unaligned 3D classifications with or without signal subtraction using masks around factor-binding sites (Supplementary Fig. 1A-C). The subtracted particle images were then reverted and subjected to 3D auto-refinement to get the final overall maps. The local resolution of the maps was improved by employing masked 3D auto-refinement using local masks around SSU-head, -body, -platform/factor region, and tail^39^. Notably, masked 3D auto-refinement failed for the SSU-head of state N1, perhaps due to high flexibility/low signal. To resolve this, the particles were exported to CryoSPARC, and masked refinement was performed using a combined mask for head-platform regions using pose/shift Gaussian priors with standard deviation 3 for rotation and 1 for shifts. The remaining masked refinements for body and tail regions for state N1 were also performed in CryoSPARC.

The maps were subjected to local resolution filtering and sharpened with a *B*-factor as reported by Relion 3.1^39^ or Relion 5.0 using appropriate solvent masks (Supplementary Fig. 1D-F). Local-mask refined, sharpened, or unsharpened maps for all states were aligned to the respective overall-mask refined map for each state and combined into a single composite map using the vopmax function in ChimeraX 1.8^40^.

In addition to mtSSU, we also observed well-defined 2D classes for mtLSU and monosome. In the RCC1L-datasets, we could identify 198 456 monosome particles and 208 368 mtLSU particles. Preliminary 3D classification of mtLSU particle sets showed distinct classes for mature mtLSU and potential assembly intermediates characterized by partial occupancy of uL11m, immature rRNA, and amorphous densities which could not be resolved (Supplementary Fig. 1A). In the NOA1-dataset, we identified distinct ~37 000 monosome particles and 156 580 mtLSU particles associated with an amorphous density. Presence of monosome in the NOA1-dataset is perhaps due to non-specific binding to the beads, which reflects in relatively much lower particle number. However, this provides evidence of a fully assembled and functional monosome in the NOA1 overexpression sample, supporting biochemical data (Supplementary Figs. 1C and 8). The reconstruction from mtLSU particles showed a large amorphous density. We did not explore mtLSU further in either RCC1L- or NOA1-datasets as this was beyond the scope of the present study. Additional data collection will be required to characterize in more detail the putative mtLSU assembly states observed in the RCC1L dataset.

For state M1, unbinned particles were subtracted on the unknown factor density for further 3D autorefinement. The map obtained was sharpened with an appropriate *B*-factor and used for assignment of the density.

Of note, all mtSSU states from RCC1L data were reproduced in NOA1 data. However, h44 remained immature in all states derived from NOA1 data due to NOA1 N-terminal loop bound to mS27, obstructing h44 folding (Supplementary Fig. 8B). We therefore excluded NOA1 data-derived maps corresponding to states M2-M4 and I1-I4 from further analysis. Importantly, NOA1 overexpression did not affect mitochondrial translation, monosome formation, or mitoribosomal protein levels (Supplementary Fig. 8C-E, also reported by ref. ^41^). BS3 non-crosslinked RCC1L-data suffered from preferred orientation and the predominant state M1 (17 280 particles) was identified and improved further with CryoSparc NU-Refine to produce an interpretable map (4.0 Å overall resolution). The remaining particles were not processed further owing to difficulties with 3D classification of anisotropic data.

### Model building and refinement

The models were manually built with Coot v0.9.8^42^ using published structures of human mtSSU or mtSSU assembly intermediates, ie, PDBID 8CSP^6^, 6RW4^8^, 7PNU, and 7PNZ^7^ respectively, as starting templates. The chains were first rigid-body fitted into the composite maps prepared by combining masked refined maps for each state. The chains were then subjected to real-space refinement with reference restraints generated in Coot v0.9.8. Depending on the local resolution, the models were then manipulated and edited to ensure correct agreement with the map. Specifically, for state N1, PDB 8CSP was used as the starting template for all mtSSU proteins, rRNA, and assembly factors, including NOA1, TFB1M, METTL17, and MCAT. Additionally, rRNA residues 1493-1496 and 1551-1557 of h44 were added to the model as they could be accommodated into the density. The ERAL1 model was taken from the AlphaFold database (AF-O75616), and the residues not supported by density, including the transit peptide residues 1-43, were removed. Overall, the regions that were relatively poorly resolved were rigid-body fitted and real-space refined with reference restraints as above. For states N2-N3, PDB 7PNZ was used as the template. rRNA was deleted wherever the support from density was missing. Models for NOA1 and TFB1M were taken from state N1 and manipulated to fit the density. At the nucleotide binding pocket of NOA1, we observed a weak but distinct density against which we could model a molecule of GDP (Supplementary Fig. 8A). In the previously published model of NOA1-mtSSU assembly complex (8CSP), the site was vacant, and the absence of GTP was attributed to an insertion that prevents Switch-I from acquiring a closed conformation^6^. There are structures of GTPases, including YqeH (bacterial homolog of NOA1), where GDP is bound in the canonical binding pocket despite a disordered switch-I, which is consistent with our interpretation (YqeH-GDP: PDBID 3EC1; EngA-GDP: PDBID 4DCU).

For state M1, we compared the maps obtained from the NOA1-dataset and the RCC1L-dataset. In both maps, M15 and PUS1 are bound to mtSSU with disordered h44, and their respective conformations are very similar; body-head orientation is comparable, and RBFA is in the IN-conformation. Importantly, we did not find any density for NOA1 N-terminus in mtSSU-tail in the map from RCC1L-dataset, which indicates that h44 distortion and PUS1 binding are likely not a result of NOA1-overexpression (Supplementary Fig. 9). With this reasoning, the map from the ‘NOA1-dataset’ with higher overall resolution (~3.1 Å) was used for model building (Supplementary Fig. 1; Supplementary Table 1). ModelAngelo^43^ was used to build a C-alpha trace into the sharpened density map. A single continuous alpha helix could be built into the density, which was supplied to “Find my sequence”^44^ as a template together with the map. The mitoproteome database was scanned and a single match, PUS1 was obtained. PUS1 model was taken from the AlphaFold Protein Structure Database^45^ (AF-Q9Y606-F1-v4) and rigid body fitted into the density, followed by restrained real space refinement. The model for RBFA was taken from PDBID 7PNU and fitted into the map with restrained real-space refinement. The rRNA h44 was built up to base-pair 1512:1537 as the density was too fragmented beyond that point for interpretation. The N-terminal loop of NOA1 (residue 68-78) was built into the density in mtSSU-tail. For states M2-4, METTL15 was taken from PDB 7PNZ, and RBFA-IN and -OUT were taken from PDB 7PNU and 7PNZ, respectively. For state M2.1, mtIF2 model was taken from PDB 6RW5. Mouse RBFA from 7PNU was mutated to human RBFA in Coot.

Ligands, metal ions, and modifications were placed based on the local density. Geometry restraints were obtained from the CCP4 7.0^46^ library or generated from the Grade Web Server (http://grade.globalphasing.org). Metal ions and waters were removed entirely or partly from the models depending on the local resolution of the maps.

The models were then hydrogenated by ReadySet in the PHENIX suite^47^. The model thus generated, together with metal ion coordination restraints, was subjected to stereochemical refinement against the respective composite density maps, wherein 4 macro-cycles of energy minimization with rotamer, Ramachandran, and reference restraints were carried out using phenix.real_space_refine^47^. Covalent bond information for methyl iso-aspartate (5F0) was manually prepared and used for real-space refinement of the models in the above step. The final models were validated with MolProbity^48^ as a part of the PHENIX suite. Refinement statistics are given in Supplementary Table 1.

### Structure analysis and visualization

Structures were analysed, and figures were generated in ChimeraX 1.8^40^. Interfaces of PUS1, METTL15, RBFA, and TFB1M reported here are solvent accessible surface areas (SASA) calculated with a simulated water molecule with a radius of 1.5 Å. Electrostatic surfaces, *B*-factor calculation, and representation were carried out using appropriate model-specific scales.

Secondary structure templates were obtained from the Ribovision suit^49^ and further adapted and stylized in Adobe Illustrator. For Supplementary Fig. 2H, the 2D topology diagram of human PUS1 was generated using PDBsum^50^ and further adapted.

### Statistics

The standard deviation is shown by error bars. A two-tailed Welch’s t-test, was used for the statistical examinations. The significance cut-off was set at *p* < 0.05, with * denoting *p* < 0.05, ** denoting *p* < 0.01, and *** denoting *p* < 0.001.

### Reporting summary

Further information on research design is available in the Nature Portfolio Reporting Summary linked to this article.

## Supplementary information

Supplementary Information

Reporting Summary

Transparent Peer Review file

## Source data

Source Data

## Data Availability

The atomic coordinates have been deposited in the RCSB PDB and the EM maps have been deposited in the Electron Microscopy Data Bank under the following accession numbers, respectively: 9H52 and EMD-51874 (State N1), 9H54 and EMD-51876] (State N2), 9H55 and EMD-51877 (State N3), 9H51 and EMD-51873 (State M1), 9ROV and EMD-54131 (State M2), 9ROT and EMD-54130 (State M2.1), 9ROR and EMD-54129 (State M3), 9RPF and EMD-54163 (State M4), EMD-57669 (State I1), EMD-57663 (State I2), EMD-57667 (State I3) and EMD-57668 (State I4). The biochemical data generated in this study are provided in the Supplementary Information and Source Data file. Source data are provided with this paper.
