# Supplementary material for: Pseudouridine synthase PUS1 and initiation factor mtIF2 are human mitoribosomal small subunit assembly factors
Source: Nat Commun. 2026 Jun 24;17:5564. doi: 10.1038/s41467-026-74700-x (PMC13294482; doi:10.1038/s41467-026-74700-x)
Supplement: Supplementary file 1 — Supplementary Information [file 41467_2026_74700_MOESM1_ESM.pdf]

SUPPLEMENTARY DATA

Pseudouridine synthase PUS1 and initiation factor mtIF2 are human mitoribosomal small subunit assembly factors

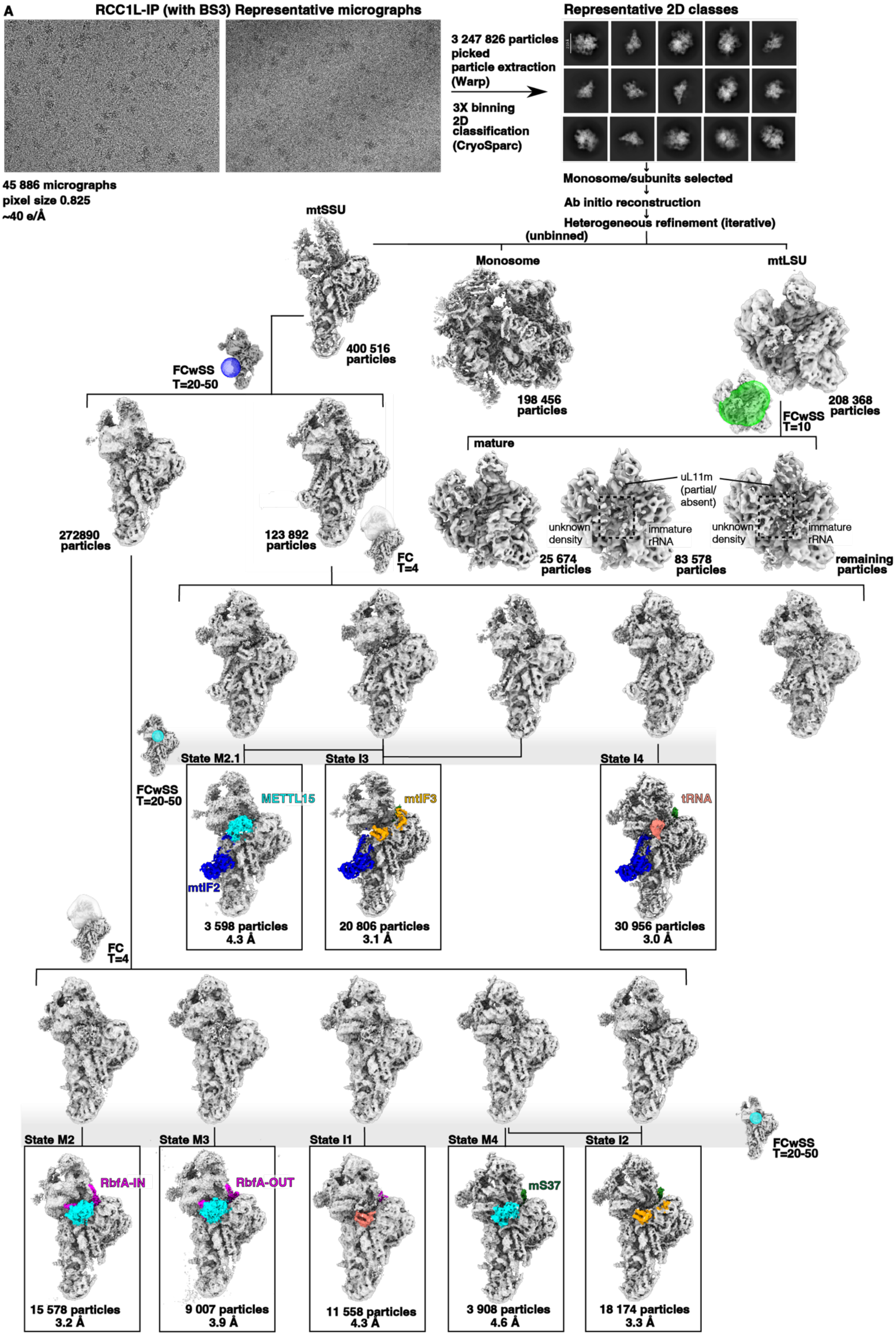
A
RCC1L-IP (with BS3) Representative micrographs
45 886 micrographs
pixel size 0.825
~40 e/Å
3 247 826 particles picked
particle extraction (Warp)
3X binning
2D classification (CryoSparc)
Representative 2D classes
Monosome/subunits selected
Ab initio reconstruction
Heterogeneous refinement (iterative)
(unbinned)
mtSSU
400 516 particles
Monosome
198 456 particles
mtLSU
208 368 particles
FCwSS T=10
mature
25 674 particles
uL11m (partial/ absent)
unknown density
immature rRNA
83 578 particles
remaining particles
FCwSS T=20-50
272890 particles
123 892 particles
FC T=4
State M2.1
METTL15
mtIF2
3 598 particles
4.3 Å
State I3
mtIF3
20 806 particles
3.1 Å
State I4
tRNA
30 956 particles
3.0 Å
State M2
RbfA-IN
15 578 particles
3.2 Å
State M3
RbfA-OUT
9 007 particles
3.9 Å
State I1
11 558 particles
4.3 Å
State M4
mS37
3 908 particles
4.6 Å
State I2
18 174 particles
3.3 Å

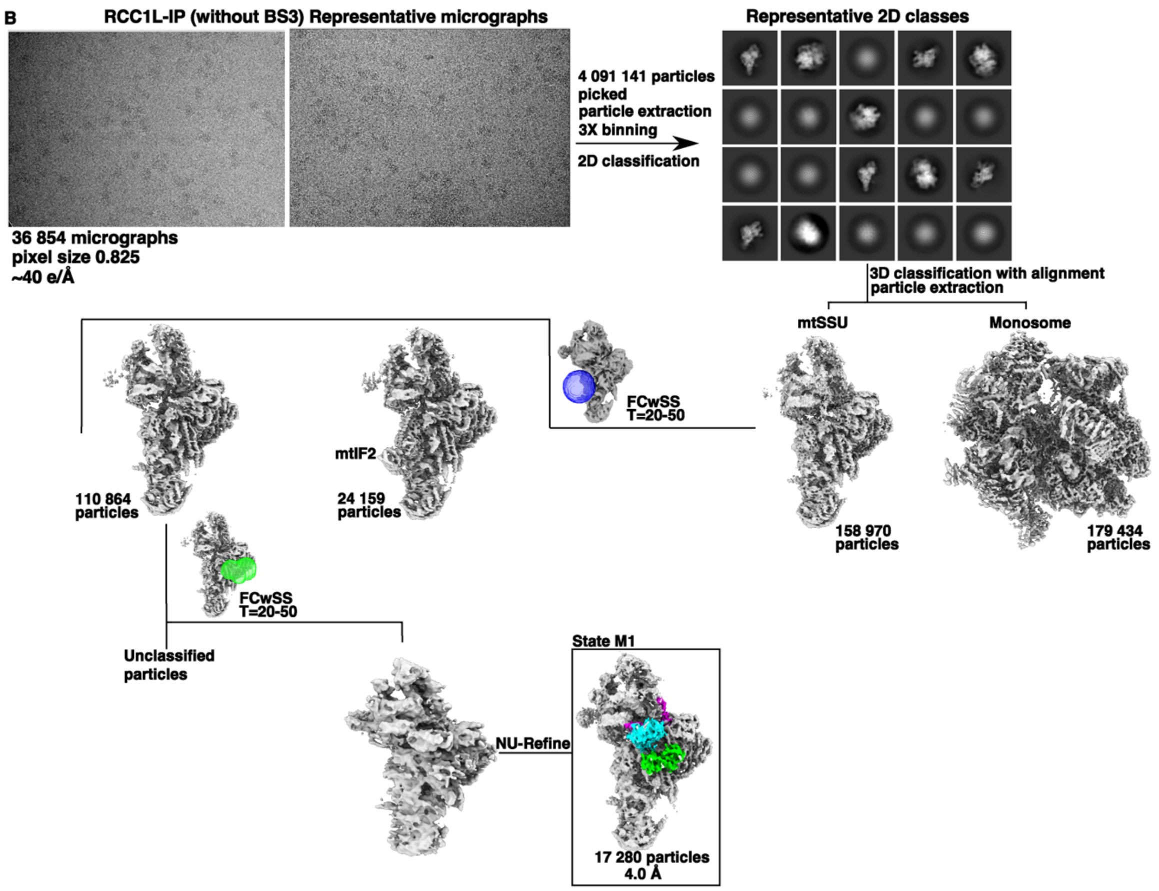
B
RCC1L-IP (without BS3) Representative micrographs
Representative 2D classes
4 091 141 particles picked
particle extraction
3X binning
2D classification
36 854 micrographs
pixel size 0.825
~40 e/Å
3D classification with alignment
particle extraction
mtSSU
Monosome
FCwSS
T=20-50
mtIF2
110 864 particles
24 159 particles
158 970 particles
179 434 particles
FCwSS
T=20-50
Unclassified particles
State M1
NU-Refine
17 280 particles
4.0 Å

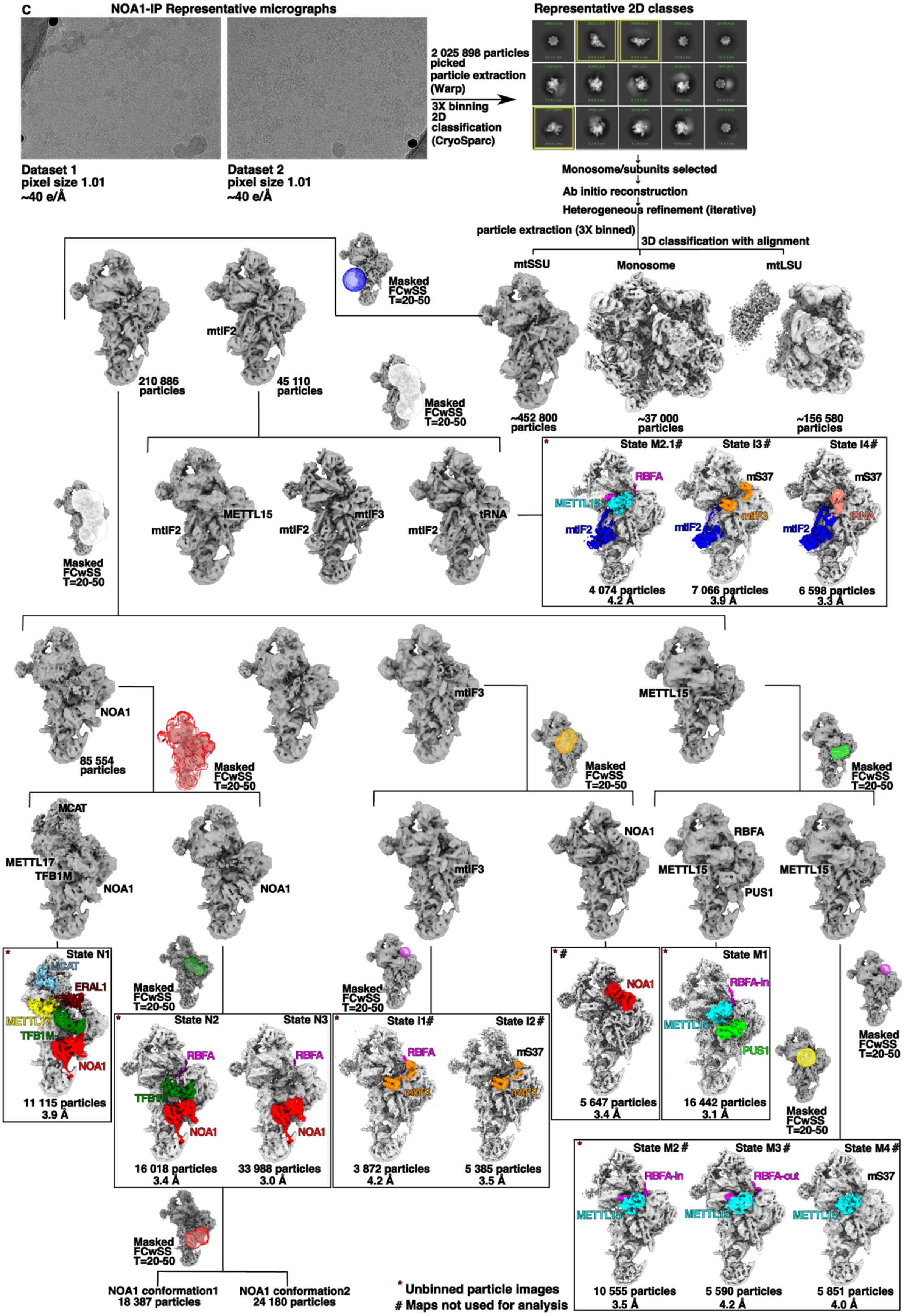
C
NOA1-IP Representative micrographs
Representative 2D classes
2 025 898 particles picked
particle extraction (Warp)
3X binning
2D classification (CryoSparc)
Dataset 1
pixel size 1.01
~40 e/Å
Dataset 2
pixel size 1.01
~40 e/Å
Monosome/subunits selected
Ab initio reconstruction
Heterogeneous refinement (iterative)
particle extraction (3X binned)
3D classification with alignment
mtSSU
Monosome
mtLSU
~452 800 particles
~37 000 particles
~156 580 particles
Masked FCwSS T=20-50
210 886 particles
mtIF2
45 110 particles
METTL15
mtIF3
tRNA
State M2.1#
State I3#
State I4#
RBFA
mS37
METTL15
mtIF3
tRNA
4 074 particles
4.2 Å
7 066 particles
3.9 Å
6 598 particles
3.3 Å
NOA1
85 554 particles
MCAT
METTL17
TFB1M
ERAL1
PUS1
State N1
11 115 particles
3.9 Å
State N2
State N3
16 018 particles
3.4 Å
33 988 particles
3.0 Å
State I1#
State I2#
3 872 particles
4.2 Å
5 385 particles
3.5 Å
5 647 particles
3.4 Å
State M1
RBFA-in
16 442 particles
3.1 Å
State M2#
State M3#
State M4#
RBFA-out
10 555 particles
3.5 Å
5 590 particles
4.2 Å
5 851 particles
4.0 Å
NOA1 conformation1
18 387 particles
NOA1 conformation2
24 180 particles
* Unbinned particle images
# Maps not used for analysis

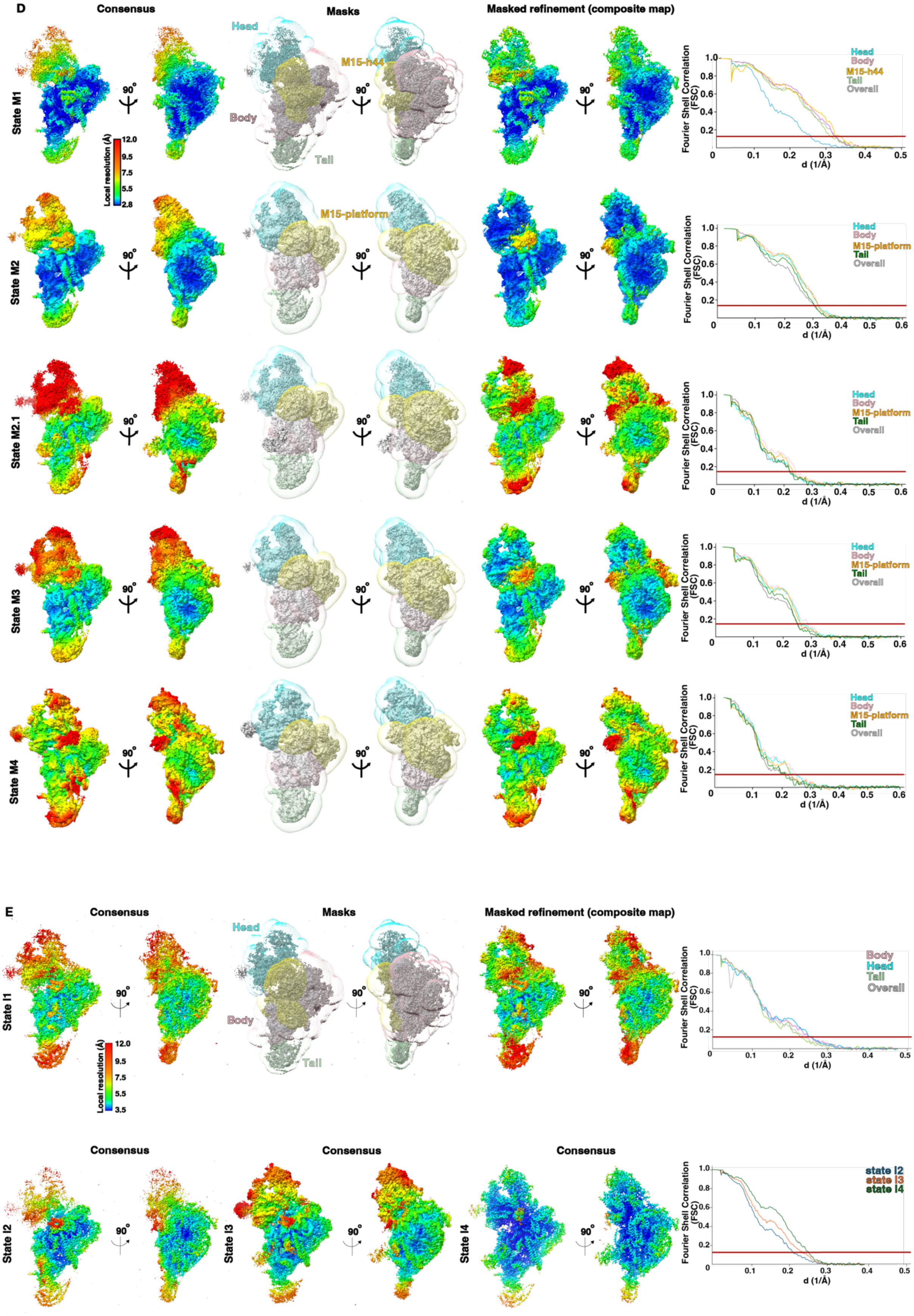
D
Consensus
Masks
Masked refinement (composite map)
Head
M15-h44
Body
Tail
Local resolution (Å)
12.0
9.5
7.5
5.5
2.8
90°
State M1
Fourier Shell Correlation (FSC)
d (1/Å)
Head
Body
M15-h44
Tail
Overall
State M2
M15-platform
State M2.1
State M3
State M4
E
State I1
3.5
State I2
State I3
State I4
state I2
state I3
state I4

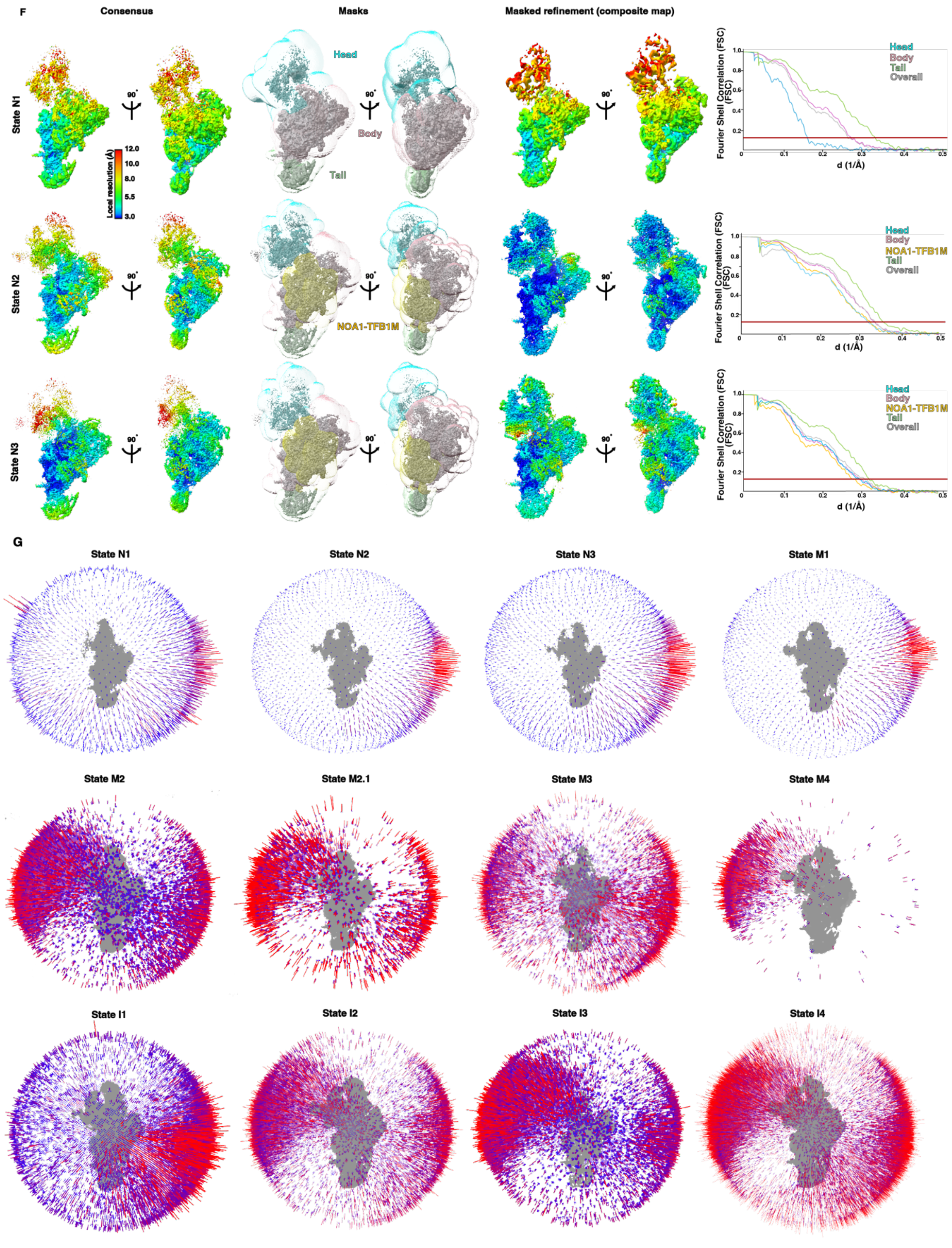

Supplementary Fig. 1. Cryo-EM data processing.

(A-C) The overview of the cryo-EM data processing scheme highlighting the 3D classification strategy employed to identify mtSSU assembly intermediates from RCC1L-IP datasets **(A,B)** and NOA1-IP datasets **(C)**.
(D-F) Local resolution maps of consensus reconstructions and of the composite maps obtained by merging masked-refined maps from the mtSSU assembly states clustered as METTL15-

containing **(D)**, initiation-factor(s)-containing classes **(E)** and NOA1-containing **(F)**. The masking scheme is shown in the central panels. Corresponding corrected FSC curves are shown where the red line marks FSC=0.143.

(G) Euler angle distribution of aligned particle images for all states, overlaid on the corresponding maps (grey).

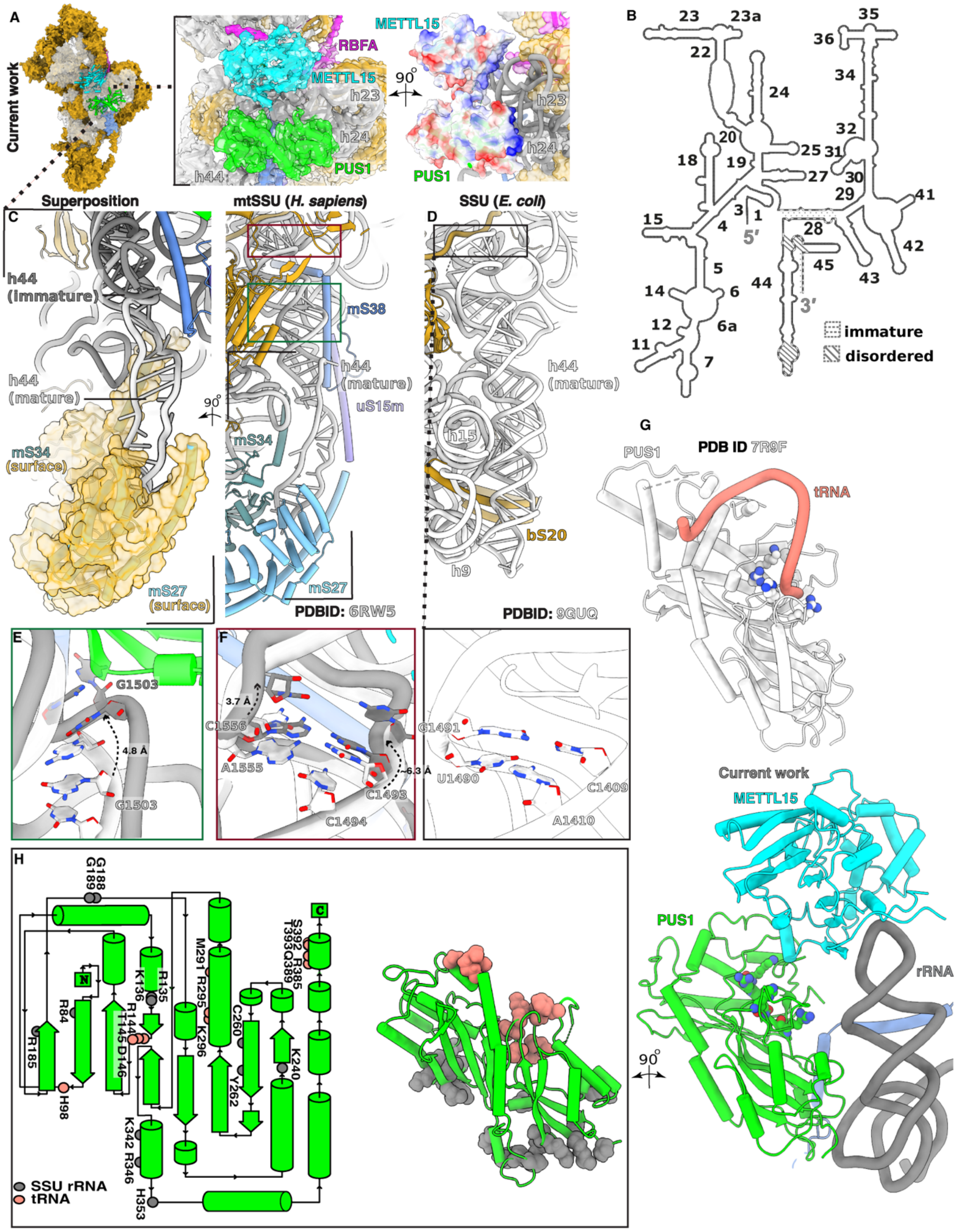

Supplementary Figure 2. Structural features of METTL15-PUS1 bound assembly intermediate.

(A) METTL15-PUS1-RBFA bound to SSU assembly intermediate together with density (colored by zone). Electrostatic potential surface of METTL15 and PUS1 form the interface with rRNA.

(B) 2D representation of 12S rRNA in this assembly state with indicated disordered regions (solid lines).

(C) Superposition (left panel) between PUS1-h44 (current work) and mature h44 (right panel) docked inside mS27 cleft (yellow surface)**.**
(D) Mature h44 in *E.coli* SSU (PDBID 9GUQ). Zoom-in panel shows base-pairs close to the decoding center (white).
(E,F) PUS1-induced conformational change in h44: **(E)** flipping-out of G1503 (dark grey) in comparison with mature h44 (light grey, transparent cartoon) is indicated; **(F)** non base-pairing residues kinked in immature h44 (current work).
(G) Comparison of PUS1-RNA complex from *S. cerevisiae* (PDB 7R9F, top) and the human PUS1-rRNA complex from the current work (bottom) Residues highlighted participate in pseudouridylation activity.
(H) The residues interacting (within 4 Å) with rRNA (grey) and tRNA (salmon; based on PDBID 7R9F). are shown in a 2D topology diagram (left) and in the model of PUS1 (right).

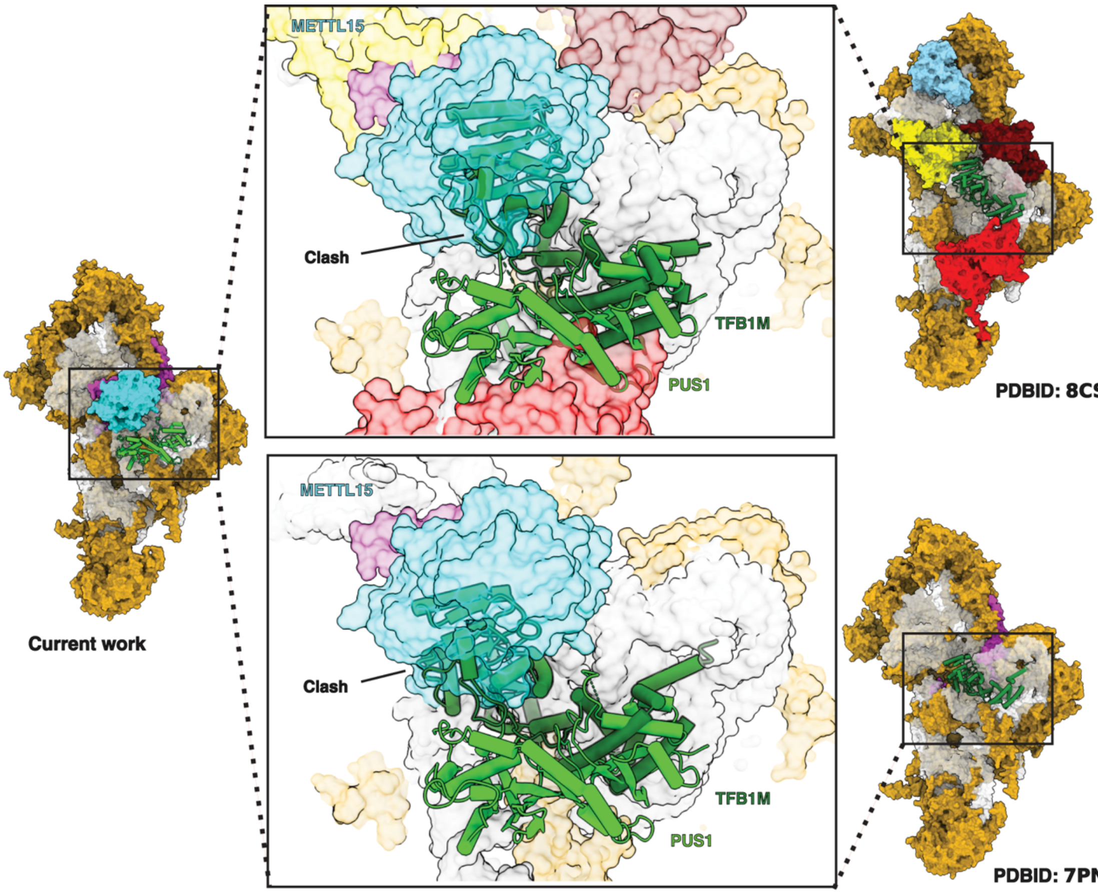

Supplementary Figure 3. Comparison between PUS1 and TFB1M bound to mtSSU assembly intermediates.

Superposition between PUS1 (light green, cartoon) and TFB1M (dark green, cartoon) in its two reported conformations (PDBID 8CSP and 7PNT). All other mtSSU components are shown in surface. METTL15 is shown in transparent surface to highlight the clash with TFB1M.

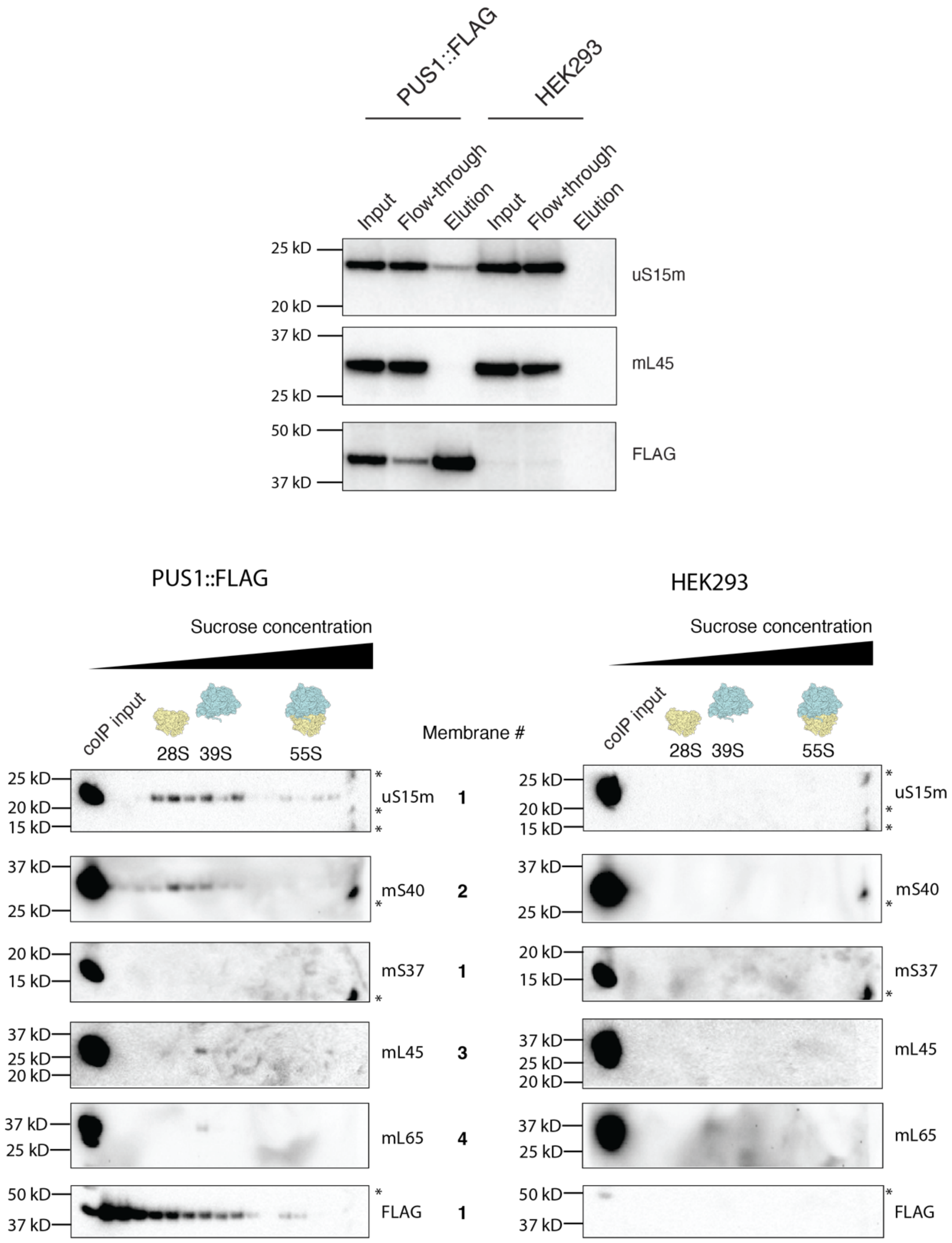

Supplementary Figure 4. Characterization of PUS1-mtSSU interactions.

Co-IP elution of PUS1::FLAG (Western blotting at the top panel, n=3) was resolved by a sucrose gradient centrifugation and probed for mitoribosome components (bottom left panel). Elution from HEK293 was used as a negative control (top and bottom right panels). Asterisk (*) indicates non-specific bands. "Membrane #" indicates the number of the membrane used for the probing. All the membranes were obtained from the sample of the same co-IP experiment. "co-IP input" lane corresponds to the "Input" sample from the co-IP experiment; its signal was adjusted to mask the oversaturated pixels. Source data are provided as a Source Data file.

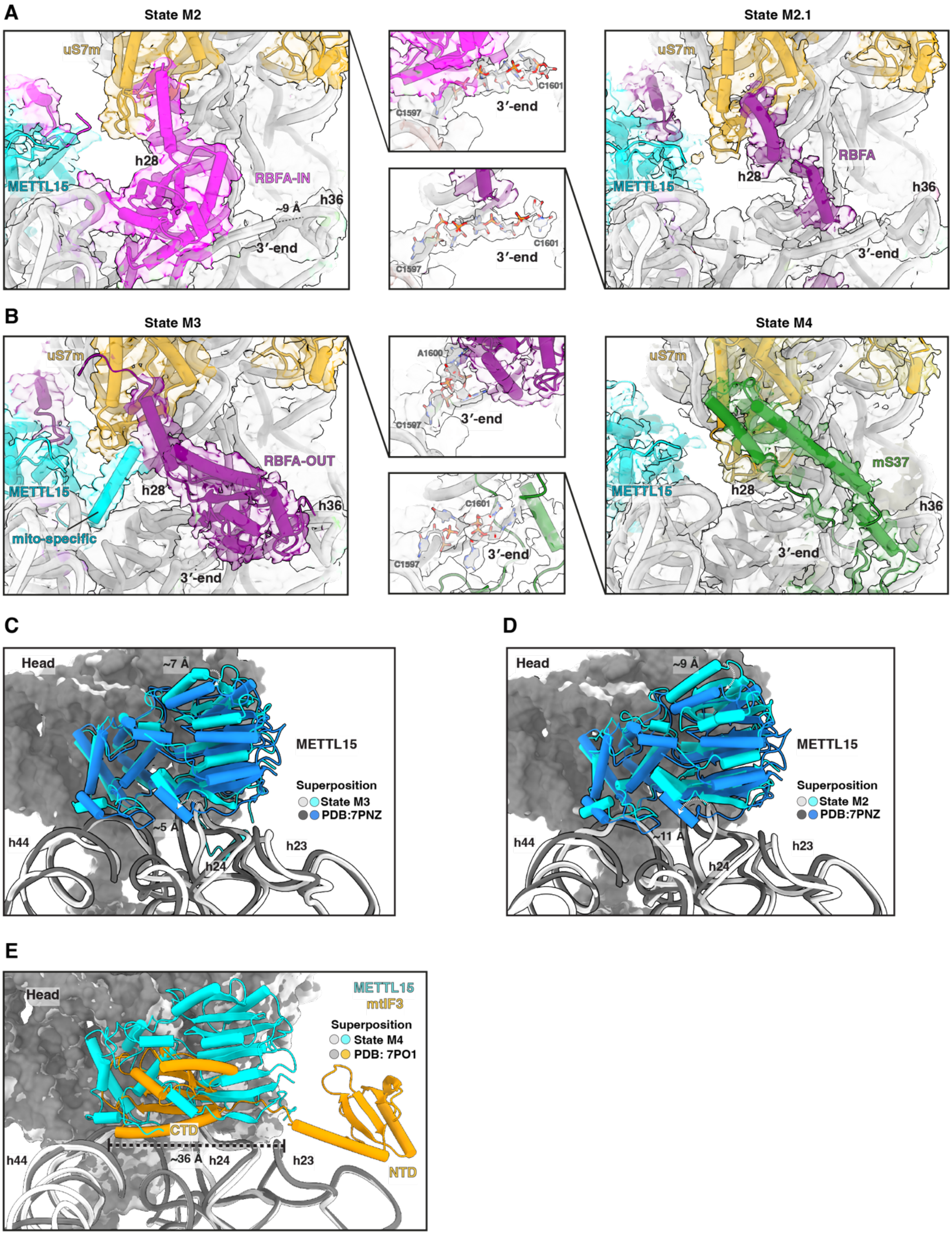

Supplementary Figure 5. 3´-end rRNA maturation and re-positioning of the platform in METTL15-bound assembly intermediates.

(A) The panels (left and right) illustrate the overall immature folding of 3´-end rRNA in states M2 and M2.1 with RBFA (magenta and purple), uS7m (gold) and METTL15 (cyan), shown with the density map. The state M2.1 signifies RBFA in transition between IN and OUT (purple)

conformation. The 3´-end rRNA makes contact with the RBFA and assumes similar fold in both assembly states. The zoom-in panels show the density for the 3´-end nucleotides (nt. 1597-1600) stabilized by contacts with the RBFA, in both the states.
(B) The panels (left and right) illustrate the folding of 3´-end rRNA in states M3 and M4. In state M4, mS37 (green) substitutes RBFA. The OUT conformation of RBFA allows 3´-end rRNA to be placed between the head and the platform, where the final folding is guided by mS37. The zoom-in panels show the density of the 3´-end nucleotides (nt. 1597-1601). The 3´-end rRNA interacts with mS37 and adopts its final conformation.
(C-D) Superposition of the final METTL15-RBFA-OUT state (PDB:7PNZ) with state M3 (left) and M2 (right), reveals the overall movement of METLL15 on mtSSU correlating with the shift in the platform (h23-h24). The color code for each state is indicated in the panel alongside.
(E) Superposition of METTL15 and mtIF3 from states M4 and PDB:7PO1 shows that METTL15 and mtIF3-CTD occupy similar space on h24 and 44, spanning a distance of ~36 Å. The binding contributes to the stabilization of the platform and blocks the decoding region prior to the recruitment of P-tRNA. The color code for each state is indicated in the panel alongside.

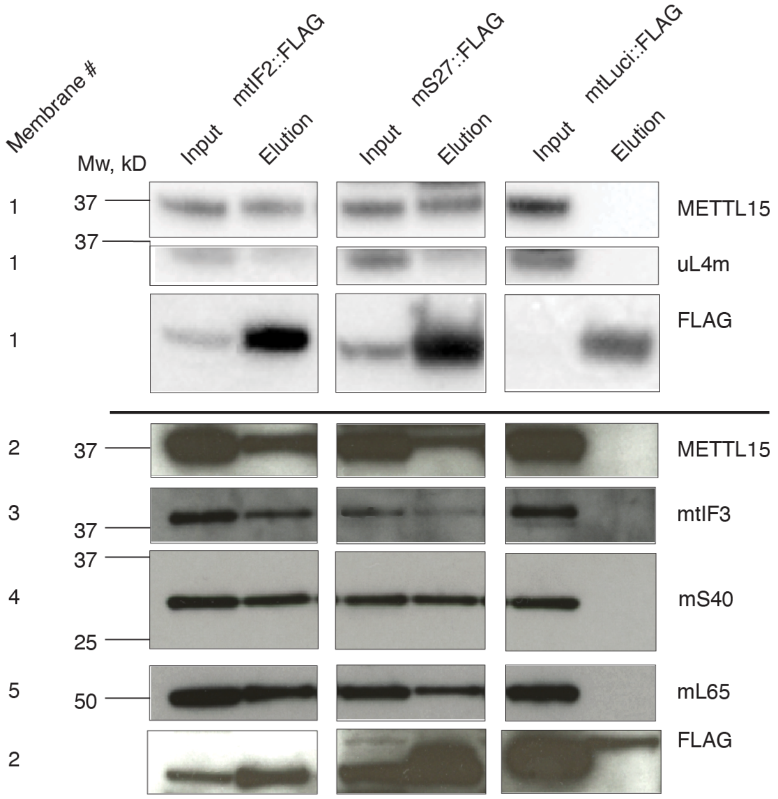

Supplementary Figure 6. Characterization of METTL15-mtIF2 interaction.

Western blot analysis of FLAG immunoprecipitation experiments (IP) using mtIF2::FLAG as bait. IP experiments with FLAG-tagged mS27 (mtSSU protein) and mitochondrially targeted luciferase (mtLuci) were used as controls. METTL15 co-precipitates with the overexpressed mtIF2::FLAG and mS27 but not mtLuci. Grey horizontal line separates the results of two independent experiments. "Membrane #" indicates the number of the membrane used for the probing. Source data are provided as a Source Data file.

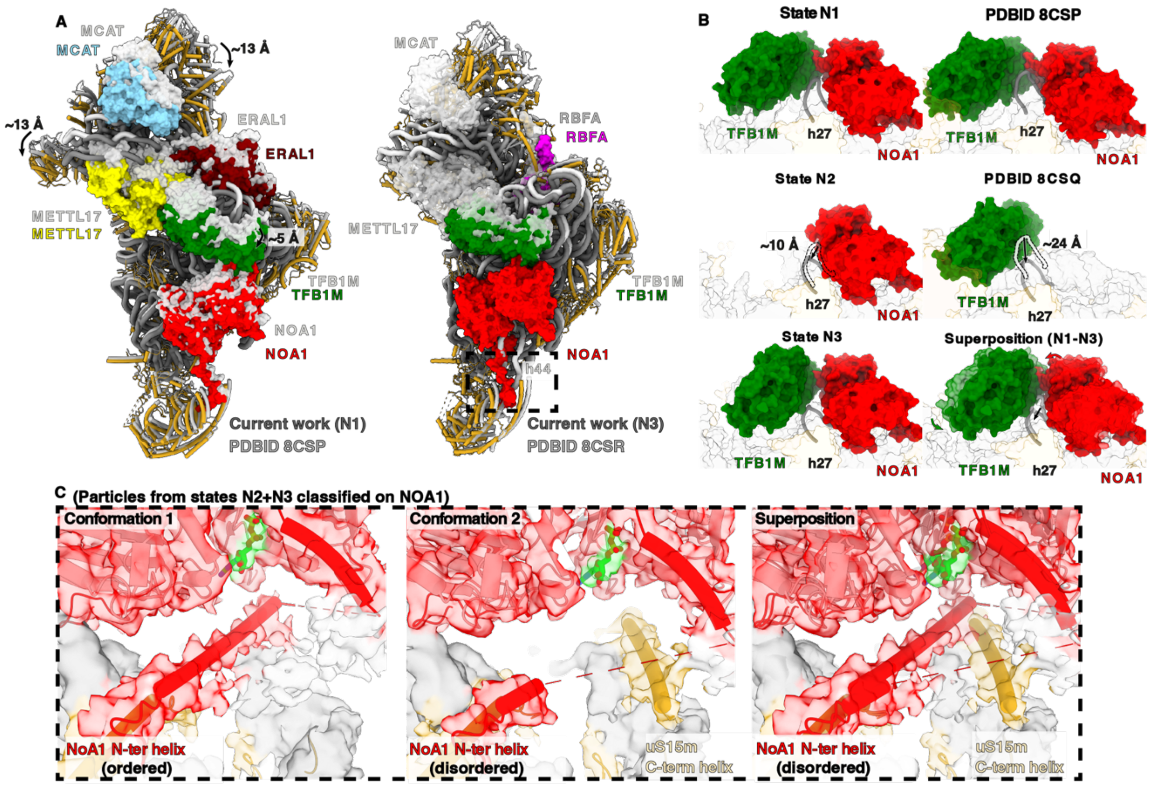

Supplementary Figure 7. Structural comparison of NOA1-bound assembly intermediates from current and previous works.

(A) Left: superposed models of state N1 (colored) and PDB 8CSP (white) show relative movement. Bound assembly factors are shown as surface. Right: superposed models of state N3 (colored) and PDB 8CSR[6] (white) highlight differences in head and body maturation.
(B) Maturation of h27 in states N1-N2 (left) versus published work[6] (right), and in state N3 (lower panel, left) together with superposition between states N1 and N3 to show the initial and final conformations of NOA1, TFB1M, and h27 (lower panel, right). Conformational changes are indicated in arrows.
(C) The zoom-in panel shows the density for the N-terminal mito-specific helix of NOA1 (states N2-N3) resolved into ordered (left) and partly disordered (center) conformations. This allows structuring of the C-terminal helix of uS15m (center). Superposition (right) reveals a steric clash between the two elements. Density is shown as a colored surface.

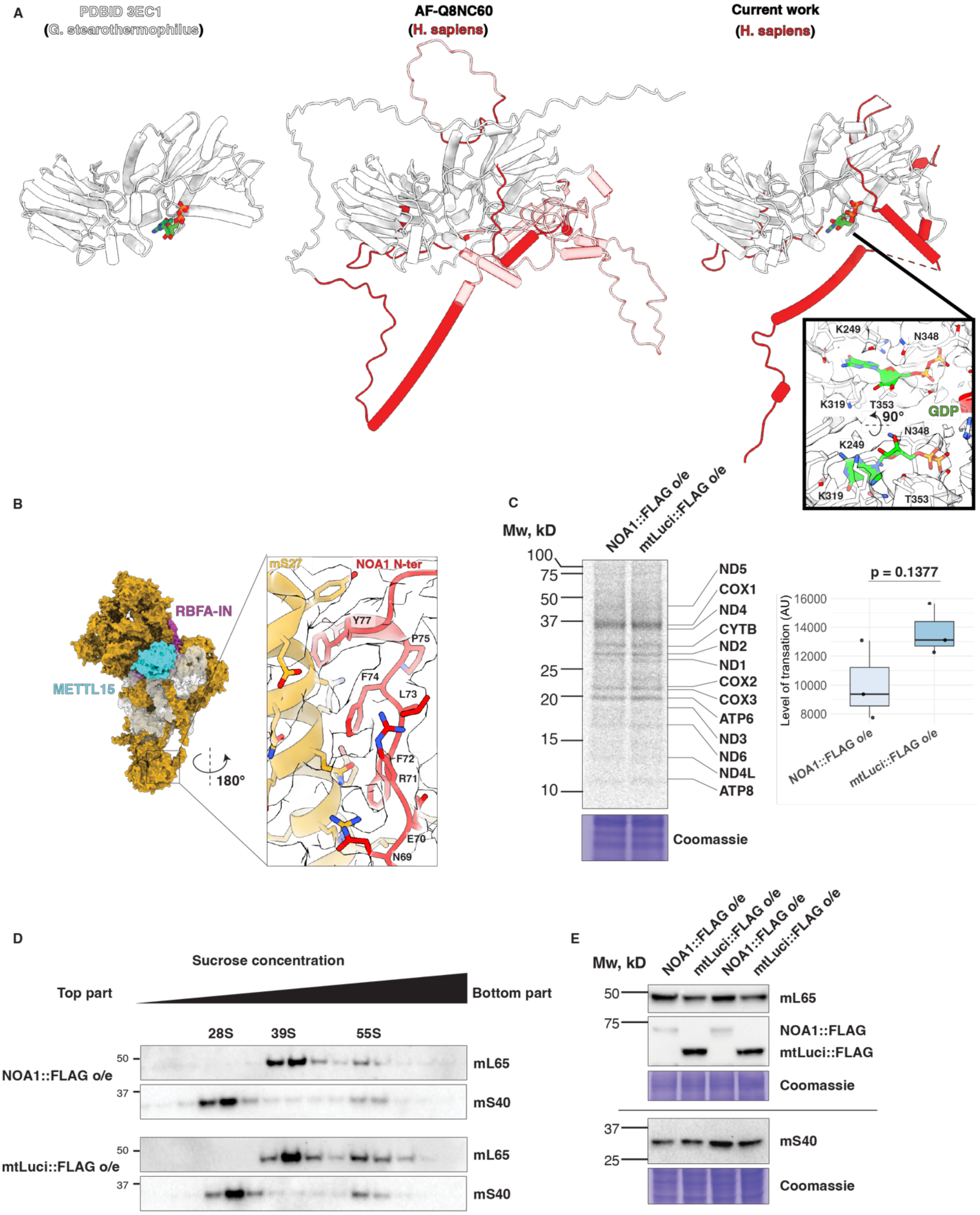

Supplementary Figure 8. NOA1::FLAG overexpression does not cause major disruptions in mitochondrial translation.

(A) Structural comparison of NOA1 from current and published work. Structure of NOA1 from *G. stearothermophilus* (PDB 3EC1, left), AlphaFold model of human NOA1 (AF-Q8NC60) and in current work. Mito-specific elements are colored red, and unmodeled regions are transparent. Zoom-in panel shows GDP binding pocket against the density map.

(B) METTL15-IN state (left) shown as the representative model to illustrate the N-terminal loop of NOA1 bound to mS27 in mtSSU tail together with the density (zoom-in). This feature is common to all 'NOA1' models reported in this work.
(**C**) Left panel: autoradiography of *de novo* labeling of mitochondrial translation with 35S-labeled methionine and cystine. FLAG-tagged mitochondrially targeted luciferase overexpression (mtLuci::FLAG o/e) was used as a control. Right panel: quantification of band intensities. The data represent mean +/-1 standard deviation from three independent experiments.
(**D**) Sucrose gradient centrifugation analysis to assess mitoribosome sedimentation patterns in cells overexpressing NOA1::FLAG compared to mtLuciferase. Mitochondrial lysates were loaded onto 10-30 % isokinetic sucrose gradients and obtained fractions were analyzed via western blotting. Membranes were probed for mtLSU: MRP mL65 and mtSSU: MRP uS40m. (n = 3 independent experiments).
(**E**) Western blotting to assess steady-state levels of MRPs (mL65 and MRP uS40m) in cells overexpressing FLAG-tagged NOA1 or mtLuciferase. Loading was determined using Coomassie staining (n = 3 independent experiments). Source data are provided as a Source Data file.

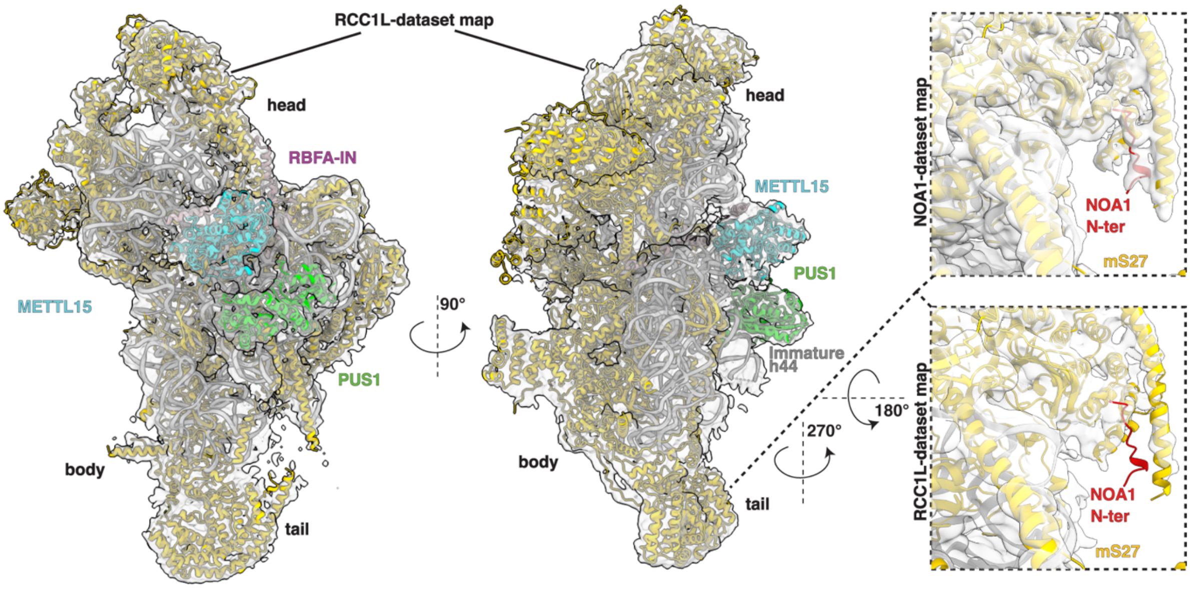

Supplementary Figure 9. Agreement of structural data between RCC1L-IP and NOA1-IP for state M1.

Superposition of state M1 map obtained from RCC1L-IP dataset with corresponding model built using map from NOA1-IP dataset shows an overall agreement for the major domains of head, body and tail; bound factors of PUS1, METTL15 and RbfA-IN; and an immature4 h44. The density for NOA1-N-ter loop observed in maps from NOA1-IP dataset is absent in that from RCC1L-IP dataset (right).

Supplementary Table 1. Cryo-EM data collection, processing and model refinement statistics

**Data collection and processing**	**state N1**	**state N2**	**state N3**	**state M1**	**state M2**	**state M2.1**	**state M3**	**State M4**	**state I1**	**state I2**	**state I3**	**state I4**
Microscope	Titan Krios	Titan Krios	Titan Krios	Titan Krios	Titan Krios	Titan Krios	Titan Krios	Titan Krios	Titan Krios	Titan Krios	Titan Krios	Titan Krios
Detector	K3	K3	K3	K3	K3	K3	K3	K3	K3	K3	K3	K3
Magnification	165,000	165,000	165,000	165,000	165,000	165,000	165,000	165,000	165,000	165,000	165,000	165,000
Voltage [kV]	300	300	300	300	300	300	300	300	300	300	300	300
Total electron exposure [$e^-/Å^2$]	42-45	42-45	42-45	42-45	42-45	42-45	42-45	42-45	42-45	42-45	42-45	42-45
Defocus range [μm]	-0.3 to -1.9	-0.3 to -1.9	-0.3 to -1.9	-0.3 to -1.9	-0.3 to -1.9	-0.3 to -1.9	-0.3 to -1.9	-0.3 to -1.9	-0.3 to -1.9	-0.3 to -1.9	-0.3 to -1.9	-0.3 to -1.9
Exposure rate ($e^-/Å^2$/sec)	20 to 25	20 to 25	20 to 25	20 to 25	20 to 25	20 to 25	20 to 25	20 to 25	20 to 25	20 to 25	20 to 25	20 to 25
Number of frames	40-42	40-42	40-42	40-42	40-42	40-42	40-42	40-42	40-42	40-42	40-42	40-42
Pixel size [Å]	0.505	0.505	0.505	0.505	0.825	0.825	0.825	0.825	0.825	0.825	0.825	0.825
Symmetry imposed	C_1	C_1	C_1	C_1	C_1	C_1	C_1	C_1	C_1	C_1	C_1	C_1
Processed particles (no.)	452,800	452,800	452,800	452,800	198,456	198,456	198,456	198,456	198,456	198,456	198,456	198,456
Final particles (no.)	11,115	33,988	16,018	16,442	15,578	3,598	9,007	3,908	11,558	18,174	20,806	30,956
Map resolution [Å] (overall)	3.9	3.0	3.4	3.1	3.2	4.3	3.9	4.6	4.3	3.3	3.1	3.0
FSC threshold	0.143	0.143	0.143	0.143	0.143	0.143	0.143	0.143	0.143	0.143	0.143	0.143
Resolution min/median/max [Å]	3.0/7.5/24.6	2.6/4.7/24.8	2.8/6.2/22.3	2.7/4.6/21.4	3.0/4.9/15.9	3.3/8.2/25.8	3.4/6.8/24.8	3.1/7.7/33.5	4.1/6.0/24.6	3.3/6.9/24.8	3.0/3.2/28.3	2.8/4.19.3
Map-sharpening *B*-factor (overall)	-80.69	-29	-29	-23	-35.3	-38.5	-37.1	-36.5	-34.7	-34.3	-31.1	-30.4
**Refinement**												
Model composition												
Total atoms (non-hydrogen/hydrogen)	128363/60614	122564/57421	129667/60765	134107/62551	128127/59502	133081/62078	127293/59156	128752/59812		-	-	-
Chains (protein/RNA)	32/1	33/1	34/1	39/1	42/1	45/1	40/1	45/1		-	-	-
RNA residues	748	803	852	926	923	905	906	930		-	-	-
Protein residues	6404	5901	6232	6357	6,007	6,370	5,988	6,028		-	-	-
Metal ions (Mg2+/K+/Zn2+)	0/0/1	42/11/1	41/10/1	40/6/1	13/0/1	24/0/1	31/0/1	2/0/1		-	-	-

Ligands (2Fe-2S/ATP/GDP/NAD/other)	2/1/1/0/1	2/1/1/0/0	2/1/1/0/0	2/1/1/1/2	2/1/2/1/2	2/1/2/1/1	2/1/1/1/1	2/1/1/1/1		-	-	-
Waters	0	0	0	3	8	9	5	8		-	-	-
Model to map CC (CC_{mask}/CC_{box}/CC_{peaks}/CC_{volume})	0.63/0.79/0.57/0.61	0.76/0.85/0.73/0.77	0.81/0.86/0.76/0.81/0.77	0.75/0.80/0.71/0.74	0.79/0.84/0.76/0.80	0.70/0.79/0.64/0.71	0.70/0.76/0.63/0.71	0.72/0.79/0.65/0.72		-	-	-
Resolution [Å] by model-to-map FSC, threshold 0.50 (masked/unmasked)	4.1/4.1	3.1/3.4	3.2/3.7	3.3/3.3	3.1/3.6	3.8/6.9	3.5/4.4	3.9/6.7		-	-	-
Average *B*-factor (RNA/protein/metal ion and ligand/water)	154/126/182/-	157/111/106/-	146/155.9/114/-	112/152/84/83	88/122/71/87	149/195/124/160	100/149/87/103	140/186/132/167		-	-	-
R.m.s. deviations, bond lengths [Å]/bond angles [°]	0.003/0.490	0.003/0.457	0.002/0.408	0.003/0.458	0.002/0.436	0.002/0.443	0.002/0.438	0.002/0.432		-	-	-
**Validation**										-	-	-
Clash score	3.39	3.31	3.92	2.58	2.32	2.24	2.69	2.07		-	-	-
Rotamer outliers [%]	0	0	0	0	0	0	0	0		-	-	-
Ramachandran plot [%] (favored/allowed/disallowed)	97.07/2.93/0.00	98.95/1.01/0.03	98.34/1.65/0.02	98.37/1.63/0.00	97.03/2.9/0.07	96.24/3.68/0.08	96.92/3.07/0.02	97.75/2.23/0.02		-	-	-
CaBLAM outliers [%]	0.98	0.7	0.89	0.62	1.03	1.13	1.13	0.82		-	-	-
C_{β} outliers [%]	0	0	0	0	0	0	0	0		-	-	-
MolProbity score	1.29	1.12	1.18	1.04	1.18	1.25	1.24	1.03		-	-	-
PDB/EMDB accession code	9H52/EMD-51874	9H54/EMD-51876	9H55/EMD-51877	9H51/EMD-51873	9IGU/EMD-52858	9IGT/EMD-52857	9IGV/EMD-52859	9RPF/EMD-54163	NA/EMD-57669	NA/EMD-57663	NA/EMD-57667	NA/EMD-57668

Supplementary Table 2. Reagents and resources

REAGENT or RESOURCE	SOURCE	IDENTIFIER
Antibodies		
mL65	Thermo Fisher Scientific	(Thermo Fisher Scientific, Cat# PA5-51245, RRID:AB_2636693)
mS40	Proteintech	(Proteintech, Cat# 16139-1-AP, RRID:AB_2146368)
anti-FLAG	Abcam	(Abcam Cat# ab205606, RRID:AB_2916341)
METTL15	Abcam	(Abcam Cat# ab307819, RRID:AB_3105766)
uL4m	Proteintech	(Atlas Antibodies, Cat# HPA051261, RRID:AB_2681413)
mtIF3	Proteintech	(Proteintech, Cat# 14219-1-AP, RRID:AB_10638621)
uS15m	Proteintech	(Proteintech Cat# 17006-1-AP, RRID:AB_2301068)
mL45	Proteintech	(Proteintech Cat# 15682-1-AP, RRID:AB_2146065)
mS37	Thermo Fisher Scientific	(Thermo Fisher Scientific Cat# PA5-58635, RRID:AB_2639747)
Anti-rabbit IgG F(ab')$_2$-HRP	GE Healthcare	(Thermo Fisher Scientific Cat# 10710965, RRID:AB_772191)
Experimental models: Cell lines		
HEK293_WT	Invitrogen	Cat. No R78007
HEK293_NOA1-FLAG_overexpression	This work	
HEK293_mtLuci-FLAG_overexpression	This work	
HEK293_mtIF2-FLAG_overexpression	This work	
HEK293_mS27-FLAG_overexpression	This work	
HEK293_PUS1-FLAG_overexpression	This work	
Oligonucleotides		
NOA1 cloning forward		CTTTCTTCTTAAGCCACCATGCTGCCCGCTCGCCTACCGTTC
NOA1 cloning reverse		CTTTCTTCTCGAGTCACTACTTATCGTCGTCATCCTTGTAATCGCTTCCTACATTTATCTTTCCTTTCTTCTTCC
RCC1L cloning forward		CTTTCTTCTTAAGCCACCATGGCGCTGGTGGCGTTGGTGGCTGG
RCC1L cloning reverse		CTTTCTTCTCGAGTTACTACTTATCGTCGTCATCCTTGTAATCGCTTCCGATGAATGACTTGGCCAGGG
Recombinant DNA		

pcDNA5/FRT/TO-NOA1::FLAG	This paper	cDNA ID: CCDS3510.1
pcDNA5/FRT/TO-RCC1L::FLAG	This paper	cDNA ID: CCDS5577.1
pcDNA5/FRT/TO-mtIF2::FLAG	GenScript Corporation	cDNA ID: CCDS1853.1
