# Supplementary material for: Pseudouridine synthase PUS1 and initiation factor mtIF2 are human mitoribosomal small subunit assembly factors
Source: Nat Commun. 2026 Jun 24;17:5564. doi: 10.1038/s41467-026-74700-x (PMC13294482; doi:10.1038/s41467-026-74700-x)
Supplement: Supplementary file 2 — Reporting Summary [file 41467_2026_74700_MOESM2_ESM.pdf]

nature portfolio

Corresponding author(s): Anas Khawaja, Joanna Rorbach

Last updated by author(s): Apr 21, 2026

Reporting Summary

Nature Portfolio wishes to improve the reproducibility of the work that we publish. This form provides structure for consistency and transparency in reporting. For further information on Nature Portfolio policies, see our Editorial Policies and the Editorial Policy Checklist.

Statistics

For all statistical analyses, confirm that the following items are present in the figure legend, table legend, main text, or Methods section.

n/a	Confirmed	
☐	☒	The exact sample size (*n*) for each experimental group/condition, given as a discrete number and unit of measurement
☐	☒	A statement on whether measurements were taken from distinct samples or whether the same sample was measured repeatedly
☐	☒	The statistical test(s) used AND whether they are one- or two-sided<br>*Only common tests should be described solely by name; describe more complex techniques in the Methods section.*
☒	☐	A description of all covariates tested
☒	☐	A description of any assumptions or corrections, such as tests of normality and adjustment for multiple comparisons
☐	☒	A full description of the statistical parameters including central tendency (e.g. means) or other basic estimates (e.g. regression coefficient) AND variation (e.g. standard deviation) or associated estimates of uncertainty (e.g. confidence intervals)
☐	☒	For null hypothesis testing, the test statistic (e.g. *F*, *t*, *r*) with confidence intervals, effect sizes, degrees of freedom and *P* value noted<br>*Give P values as exact values whenever suitable.*
☒	☐	For Bayesian analysis, information on the choice of priors and Markov chain Monte Carlo settings
☒	☐	For hierarchical and complex designs, identification of the appropriate level for tests and full reporting of outcomes
☒	☐	Estimates of effect sizes (e.g. Cohen's *d*, Pearson's *r*), indicating how they were calculated

Our web collection on statistics for biologists contains articles on many of the points above.

Software and code

Policy information about availability of computer code

Data collection	Western blot data were collected using either a ChemiDoc XRS+ system (Bio-Rad) or Amersham Hyperfilm MP films (Cytiva)<br><br>De novo mitochondrial translation data were collected on Typhoon FLA 7000 Phosphorimager (GE Healthcare).<br><br>Cryo-EM data were collected with a Krios G3i electron microscope (Thermofisher) operated at 300 kV and equipped with a K3 Bioquantum detector (Gatan). Automated data collection software was used during collection (EPU 2, Thermofisher). Collection was performed at 165,000x (105,000x) EFTEM SA magnification, yielding calibrated pixel sizes of 0.507 Å for NOA1 and 0.825 Å for RCC1L datasets.
Data analysis	Image analysis was performed in Imagej 1.53t.<br><br>Statistical analyses were carried out in Python (3.9.21) and SciPy (1.13.1)<br><br>For cryoEM data, Motion correction, CTF-estimation, Fourier cropping (to 1.015Å/px for NOA1 data), picking and extraction in 600 pixel boxes were performed on the fly using WARP 4.0. Cryosparc v4 was used to run 2D classification. Cryosparc v4, Relion 3.1, Relion 5.0 were used for further analyses.

For manuscripts utilizing custom algorithms or software that are central to the research but not yet described in published literature, software must be made available to editors and reviewers. We strongly encourage code deposition in a community repository (e.g. GitHub). See the Nature Portfolio guidelines for submitting code & software for further information.

Data

Policy information about availability of data

All manuscripts must include a data availability statement. This statement should provide the following information, where applicable:

- Accession codes, unique identifiers, or web links for publicly available datasets
- A description of any restrictions on data availability
- For clinical datasets or third party data, please ensure that the statement adheres to our policy

The atomic coordinates have been deposited in the RCSB PDB and the EM maps have been deposited in the Electron Microscopy Data Bank under the following accession numbers, respectively: 9H52 and EMD-51874 (State N1), 9H54 and EMD-51876 (State N2), 9H55 and EMD-51877 (State N3), 9H51 and EMD-51873 (State M1), 9ROV and EMD-54131 (State M2), 9ROT and EMD-54130 (State M2.1), 9ROR and EMD-54129 (State M3), 9RPF and EMD-54163 (State M4), EMD-57669 (State I1), EMD-57663 (State I2), EMD-57667 (State I3) and EMD-57668 (State I4).

Research involving human participants, their data, or biological material

Policy information about studies with human participants or human data. See also policy information about sex, gender (identity/presentation), and sexual orientation and race, ethnicity and racism.

Reporting on sex and gender	N/A
Reporting on race, ethnicity, or other socially relevant groupings	N/A
Population characteristics	N/A
Recruitment	N/A
Ethics oversight	N/A

Note that full information on the approval of the study protocol must also be provided in the manuscript.

Field-specific reporting

Please select the one below that is the best fit for your research. If you are not sure, read the appropriate sections before making your selection.

☒ Life sciences ☐ Behavioural & social sciences ☐ Ecological, evolutionary & environmental sciences

For a reference copy of the document with all sections, see nature.com/documents/nr-reporting-summary-flat.pdf

Life sciences study design

All studies must disclose on these points even when the disclosure is negative.

Sample size	Sample size was determined based on field standards for obtaining sufficient statistical power (at least 3 biological replicates for experiments involving statistical analysis). Exact sample sizes are depicted in Figure legends.
Data exclusions	No data were excluded from the analysis.
Replication	Assessment of negative effects of NOA1:FLAG overexpression on mitochondrial translation, steady-state levels of mitoribosomal proteins, mitoribosome levels, was performed three times.<br>Characterization of METTL15-mtIF2 interaction was performed two times.<br><br>All attempts in replication were successful.
Randomization	Randomization was not required for this study, as (i) no human or animal subjects were studied, (ii) quantitative data were collected, (iii) there was no danger of confounding independent variables in the experimental design.
Blinding	Blinding was not relevant to this study. For biochemical characterization of cell lines the investigator has to be aware of the sample identity.

Reporting for specific materials, systems and methods

We require information from authors about some types of materials, experimental systems and methods used in many studies. Here, indicate whether each material, system or method listed is relevant to your study. If you are not sure if a list item applies to your research, read the appropriate section before selecting a response.

Materials & experimental systems

n/a	Involved in the study
☐	☒ Antibodies
☐	☒ Eukaryotic cell lines
☒	☐ Palaeontology and archaeology
☒	☐ Animals and other organisms
☒	☐ Clinical data
☒	☐ Dual use research of concern
☒	☐ Plants

Methods

n/a	Involved in the study
☒	☐ ChIP-seq
☒	☐ Flow cytometry
☒	☐ MRI-based neuroimaging

Antibodies

Antibodies used

All antibodies used in this study are listed in detail in Key resources table under Methods section.

antibody (manufacturer, Cat#, RRID)

mL65 (Thermo Fisher Scientific, Cat# PA5-51245, RRID:AB_2636693)
mS40 (Proteintech, Cat# 16139-1-AP, RRID:AB_2146368)
anti-FLAG (Abcam Cat# ab205606, RRID:AB_2916341)
METTL15 (Abcam Cat# ab307819, RRID:AB_3105766)
uL4m (Atlas Antibodies, Cat# HPA051261, RRID:AB_2681413)
mtIF3 (Proteintech, Cat# 14219-1-AP, RRID:AB_10638621)
uS15m (Proteintech Cat# 17006-1-AP, RRID:AB_2301068)
mL45 (Proteintech Cat# 15682-1-AP, RRID:AB_2146065)
mS37 (Thermo Fisher Scientific Cat# PA5-58635, RRID:AB_2639747)
Anti-rabbit IgG F(ab')2-HRP (Cytiva Cat# 10710965, RRID:AB_772191)

Validation

- mL65 antibody was verified in this study via Western blot:
  - band size matches molecular weight of mL65
  - position of the band on sucrose gradient matches the one the large mitoribosome subunit

- mS40 antibody was vefified by manufacturer, Proteintech via Western blot:
  - cell lysates of mammalian cell lines A549, HEK293, MCF-7, PC-3, SH-SY5Y
  - mouse liver tissue lysate
  - rat heart tissue lysate
  - co-immunoprecipitation from cell lysate of HeLa cell line

- anti-FLAG antibody was vefified by manufacturer, Abcam via Western blot:
  - cell lysate of HEK-293 cell line expressing with FLAG-tagged PFKFB3
  - co-immunoprecipitation of FLAG-tagged PFKFB3, cell lysate of HEK-293T
  - HEK293T cell lysate with transient overexpression of a number of FLAG-tagged proteins, including CLIP1/CLIP170/PNS-Tau

- anti-METTL15 antibody was vefified by manufacturer, Abcam via Western blot:
  - Human colon tissue lysate
  - Human tonsil tissue lysate
  - cell lysates of mammalian cell lines A431, HEK293T
  - cell lysate of HeLa cell line transfected with siRNA specifically targeting METTL15

- anti-uL4m antibody was vefified by manufacturer, Atlas Antibodies via Western blot:
  - cell lysates of mammalian cell lines RT-4_br, U-251MG sp

- anti-mtIF3 antibody was vefified by manufacturer, Proteintech via Western blot:
  - cell lysates of mammalian cell lines A431, HT-29, HeLa, HepG2
  - cell lysates of wild-type and MTIF3 knockout human preadipocyte cells
  - co-immunoprecipitation of mtIF3, cell lysate of HeLa

- anti-uS15m antibody was vefified by manufacturer, Proteintech via Western blot:
  - cell lysates of mammalian cell lines HeLa, MCF-7, Raji
  - mouse liver tissue lysate
  - co-immunoprecipitation of uS15m, mouse brain tissue lysate

- anti-mL45 antibody was vefified by manufacturer, Proteintech via Western blot:
  - cell lysates of mammalian cell lines HeLa, SKOV-3
  - cell lysate of HeLa cell line transfected with siRNA specifically targeting mL45

Eukaryotic cell lines

Policy information about cell lines and Sex and Gender in Research

Cell line source(s)	Flp-In TREx cell line was purchased from Thermofisher Scientific (catalog number: R78007).<br>NOA1::FLAG, PUS1::FLAG, RCC1L::FLAG, mtIF2::FLAG, mtLuciferase::FLAG and mS27::FLAG -overexpressing cell lines were generated from Flp-In TREx cell line as described in Methods.
Authentication	Generation of overexpressing cell lines: NOA1::FLAG, PUS1::FLAG, RCC1L::FLAG, mtIF2::FLAG, mtLuciferase::FLAG and mS27::FLAG was validated by western blotting (using anti-FLAG antibody).
Mycoplasma contamination	Cell lines tested negative for mycoplasma contamination.
Commonly misidentified lines<br>(See ICLAC register)	No commonly misidentified cell lines were used in the study.

Plants

Seed stocks	*Report on the source of all seed stocks or other plant material used. If applicable, state the seed stock centre and catalogue number. If plant specimens were collected from the field, describe the collection location, date and sampling procedures.*
Novel plant genotypes	*Describe the methods by which all novel plant genotypes were produced. This includes those generated by transgenic approaches, gene editing, chemical/radiation-based mutagenesis and hybridization. For transgenic lines, describe the transformation method, the number of independent lines analyzed and the generation upon which experiments were performed. For gene-edited lines, describe the editor used, the endogenous sequence targeted for editing, the targeting guide RNA sequence (if applicable) and how the editor was applied.*
Authentication	*Describe any authentication procedures for each seed stock used or novel genotype generated. Describe any experiments used to assess the effect of a mutation and, where applicable, how potential secondary effects (e.g. second site T-DNA insertions, mosiacism, off-target gene editing) were examined.*
